# Genetic coping mechanisms observed in *Leishmania tropica*, from the Middle East region, enhance the survival of the parasite after drug exposure

**DOI:** 10.1371/journal.pone.0310821

**Published:** 2024-12-03

**Authors:** Hedvig Glans, Gabriel M. Matos, Maria Bradley, Tim Downing, Björn Andersson

**Affiliations:** 1 Department of Infectious Diseases, Karolinska University Hospital, Stockholm, Sweden; 2 Division of Dermatology and Venerology, Department of Medicine Solna, Karolinska Institutet, Stockholm, Sweden; 3 Department of Cell and Molecular Biology, Karolinska Institutet, Stockholm, Sweden; 4 School of Biotechnology, Dublin City University, Dublin, Ireland; 5 The Pirbright Institute, Woking, United Kingdom; Kerman University of Medical Sciences, ISLAMIC REPUBLIC OF IRAN

## Abstract

**Introduction:**

Cutaneous leishmaniasis caused by *L*. *tropica* is common in the Middle East and treatment failure and drug resistance are known to occur. Several genetic mechanisms: aneuploidy, recombination and loss of heterozygosity, single nucleotide polymorphism (SNP) changes, copy number variation (CNV), and mutation of the H locus associated with drug resistance have been described.

**Materials and methods:**

We studied SNP and CNV patterns in 22 isolates of *L*. *tropica* from Afghanistan, Iran and Syria in a geographic, phylogenetic and antimony exposure context.

**Results:**

A high SNP frequency was observed in isolates from Syria on chromosome 23, including the H locus, linked to different ancestry at that chromosome segment. Among the isolates from Afghanistan and Iran, an elevated frequency of nonsynonymous SNPs was observed on several chromosomes. Changes in CNV patterns were seen in isolates exposed to drug pressure, especially for the ferric iron reductase gene. Expanded genes were categorised into five functional categories: translational elongation, mitochondrial transmembrane transport, positive regulation of cellular component organisation, response to stimulus and response to hypoxia. No CNV was identified at the H locus, the MAPK1 gene, the APQ1 gene, nor chromosomes 23, 31 or 36 regardless of previous antimonial exposure.

**Discussion:**

In our study, *Leishmania tropica* had a jump in the nonsynonymous SNP rates at chromosome 23, including the H locus. CNV was observed among isolates exposed to antimonials, especially involving the gene encoding a ferric iron reductase. Several essential genetic coping mechanisms in the cell were enhanced when exposed to antimony, possibly for the survival of the parasite. Our work supports the perspective that *Leishmania* uses several mechanisms to adapt to environmental changes and drug exposure.

## Introduction

Leishmaniasis is a neglected tropical infection caused by the parasite *Leishmania*. Millions of people are affected in endemic regions worldwide [1]. Different *Leishmania* spp. are associated with different clinical manifestations; cutaneous leishmaniasis (CL), mucocutaneous leishmaniasis and visceral leishmaniasis (VL) [2]. Clinical manifestations, treatment and outcome are affected by factors like the host genetic variability, host immune response, environmental factors and *Leishmania* genetic variation [3]. *L*. *tropica* causes CL across Africa and the Middle East [1, 4, 5]. Due to local conflicts and associated population displacement, migration and relocation, the number of *L*. *tropica* cases is increasing in several regions [1]. In the Middle East, CL caused by *L*. *tropica* is characterised by relapses (leishmaniasis recidivans) and difficulties in treatment, often multiple and prolonged treatments are necessary [6, 7]. *L*. *tropica* uses different transmission mechanisms, both zoonotic and anthroponotic [8, 9], which could affect drug resistance [10]. The anthroponotic nature of the organism and genetic mechanisms linked to drug resistance motivates identifying determinants associated with drug resistant, anthroponotic *L*. *tropica* to reduce the spreading [10].

### Adaptation to environmental changes

The *Leishmania* genome shows frequent mosaic aneuploidy, copy number variation (CNV) of genes and extrachromosomal circular or linear amplification of sets of genes [11–15]. These have been hypothesised to be mechanisms for adaptation to environmental changes, and to compensate for the lack of transcriptional regulation [16, 17].

The mechanisms are not fully understood, but genome plasticity, such as CNVs in combination with intrinsic genetic factors that generate strain-specific phenotypes is important [12, 18–23].

An amplification of gene copy number and increased activity of proteins involved in drug removal has been associated with drug resistance in *Leishmania* [21, 24–26]. A variation of somy and aneuploidy are seen early during *in vivo* drug pressure and later SNPs, indels or gene deletions have been described [21, 27, 28]. Aneuploidy and gene copy number are both used to quickly increase or decrease the gene dosage, i.e. a gain- or loss of function [27], and this makes the parasite more adaptive and sensitive to environmental changes [29] and stress [21]. Somy changes often emerge rapidly in response to such events [13, 20], but the karyotypic optima vary among genetically related samples [19]. CNV cause a genomic and phenotypic heterogeneity that cause the interaction between the parasite’s cellular invasion and the host’s immune system [15].

### Gene dosage

*L*. *donovani* [13, 30], *L*. *tropica* [18] and *L*. *infantum* [23] demonstrated several adaptive mechanisms during drug pressure to modulate gene dosage of therapeutic targets and other determinants of resistance. Generation of episomal amplicons [22], changes in ploidy, local gene CNV, SNPs at genes encoding drug targets, or the upregulation of proteins seem to be involved in intracellular survival of the parasite [31]. Increased allelic variation due to CNV at drug target or transporter genes can lead to drug resistance but may not affect the gene expression [32]. SNPs in *L*. *donovani* isolates from the Indian subcontinent have caused antimonial resistance [13]. Similar genetic mechanisms were identified in sequenced *L*. *braziliensis* and *L*. *panamensis* genome data before and after treatment with antimony. This indicated that several different genetic mechanisms may be involved in *Leishmania* spp. under antimonial pressure during treatment [31].

The patterns and extent of gene amplification vary between *Leishmania* spp. and geographic regions [19, 33]. Genetically related but allopatric *Leishmania* isolates can be distinguished by both the amplification and loss of genes. This could be a result of different selection processes and the adaptation of *Leishmania* to a given environment [19]. The same picture has been seen for the susceptibility to antimony among different *Leishmania* populations globally [33]. Varied results in treatment response and outcome have been described in different geographic *Leishmania* populations [34]. Previous work analysing different *L*. *donovani* populations observed several sodium stibogluconate resistant (SSG-R) strains possessing several independent genetic mechanisms of drug resistance, all of which spread over time [13]. Moreover, the discovery of recombinant SSG-R linages highlights the surprising redundancy and plasticity of these adaptive processes [13, 19].

### Mechanisms of drug resistance

A number of suggested genetic mechanisms correlated with antimony-resistance have been described: decreased cellular entry, decreased drug activation/reduction, and increased efflux and sequestration of the metal-thiol conjugate into intracellular vesicles [35–40].

Mutations in genes can alter drug function and efficacy [41], the amplification of genes can increase gene expression, and somy changes can modify the effect of the drug [21, 33]. A key component of the parasite drug response is the H-locus, which has been associated with drug resistance and can be involved in the efflux of antimony in *Leishmania* (S1 Table) [21, 42–44].

Other than the H-locus, the mitogen-activated protein kinase 1 (MAPK1) gene [45] is involved in drug resistance [33]: we know that MAPK1 gene expression changes affect drug treatment outcome [45, 46]. Furthermore, an increased copy number of the MAPK 1 gene in isolates from patients with VL and HIV correlated with drug resistance after repeated treatment [42], perhaps exacerbated by long-term exposure to heavy metals similar to antimonials [30, 33]. Another locus associated with drug resistance is the aquaglyceroporin 1 (AQP1) gene, at which a homozygous insertion conferred resistance [43]. An overexpression of trypanothione biosynthesis genes may also play a role in antimony resistant of anthroponotic *L*. *tropica* [47].By comparing 22 sequenced *L*. *tropica* isolates from Afghanistan, Iran and Syria, we studied CNV and SNP variability between isolates exposed to antimonial prior sampling (treatment failures) and isolates not exposed to antimony. Our results provide information on genetic mechanisms *Leishmania tropica* uses to adapt to environmental changes during infection and drug pressure.

## Material and methods

### Ethical statement

Ethical approval was obtained from the Central Ethical Review Board in Stockholm (2015/2162–31, 2017/121-32). The Ethical committee waived the requirement for informed consent as the samples, included in the genome study, and the clinical data were anonymized, and only isolated parasites were studied. Clinical data were collected during 2017–2018, by one researcher.

### Sample collection

Individual *L*. *tropica* isolates were sampled from 21 patients diagnosed with CL between 2007–2017. The patients were from Afghanistan (n = 3), Iran (n = 2) and Syria (n = 17). One patient, from Syria, with leishmaniasis recidivans, was sampled twice, three years apart (14_01223 and 17_01604) after several treatment failures and relapses when treated with antimonial, liposomal amphotericin and cryotherapy. Data previously published in [48]. In total we used 22 *Leishmania* genomes for investigation.

The isolates were divided into two groups, A and B, depending on antimonial treatment prior to sampling (Table 1).

**Table 1:** The 22 isolates are divided into two groups.

Sample ID	Group	Country	Antimonial treatment prior to sampling	Genetic group	Treatment after sampling	Treatment outcome
07_00242	A	Iran	Yes, antimonial	Reference	No treatment	Cured
13_00550	A	Syria	Yes, antimonial	Reference	Antimony	Cured
13_01390	A	Syria	Yes, antimonial	Reference	Antimony	Relapse
14_00771	A	Syria	Yes, antimonial	Non-reference	LA	Cured
14_01223^a^	A	Syria	Yes, antimonial	Non-reference	Antimony	Treatment failure
15_00019	A	Syria	Yes, antimonial	Non-reference	LA	NA
15_01088	A	Syria	Yes, antimonial	Non-reference	Antimony	Cured
16_14706	A	Syria	Yes, antimonial	Non-reference	Cryotherapy	Cured
17_01604^a^	A	Syria	Yes, antimonial	Non-reference	NA	NA
15_02597	B	Syria	No	Non-reference	Cryotherapy	Treatment failure
07_01513	B	Syria	No	Reference	NA	NA
13_01024	B	Syria	No	Non-reference	Cryotherapy	Cured
13_01233	B	Afghanistan	No	Reference	Cryotherapy	Cured
14_00642	B	Syria	No	Reference	Antimony	Relapse
14_00849	B	Syria	No	Non-reference	Antimony	Cured
15_01620	B	Syria	No	Reference	LA	Treatment failure
15_02015	B	Syria	No	Non-reference	Antimony	Cured
15_02480	B	Afghanistan	No	Non-reference	Fluconazole	Treatment failure
15_02576	B	Syria	No	Non-reference	Cryotherapy	Cured
16_00075	B	Afghanistan	No	Non-reference	LA + Cryotherapy	Treatment failure
16_00674	B	Syria	No	Non-reference	LA	Cured
16_00964	B	Iran	No	Non-reference	Antimony + Cryotherapy	Cured

Group A had a history of antimonial treatment prior to sampling and the lesion had not healed, treatment failure [49]. Group B had no history of any antimonial treatment. Twelve were cured after treatment, but only eight were treated with antimonial [48]. Each isolate is also divided after the genetic correlation to reference genome, non-reference and reference. ^a^ two isolates from the same patient. LA–liposomal amphotericin B, NA–not available.

Group A had received antimonial treatment prior to sampling but had a treatment failure (nine patients) and group B had not been exposed to any antimonial treatment (13 patients). Treatment failure was defined as the absence of re-epithelialisation in the lesion during or within 2 months after treatment, and relapse, the recurrence of a previously healed lesion without new exposure within 12 months after treatment started [49]. Isolate 15_02597 received cryotherapy, but no chemotherapy prior, to sampling and was included in group B. Within each group, the isolates were divided into two subgroups, after genetic groups and correlation to reference genome [48] to reduce variation due to phylogenetic differences.

All patients but one, who disappeared after sampling, were followed-up and received treatment. Different treatments were used; antimonial, liposomal amphotericin B, cryotherapy and fluconazole (Table 1). Data previously published in [48]. In group B, four received antimonial treatment. Three healed their lesions and one relapsed.

Culturing, DNA extraction, library preparation and short-read sequencing were performed as described in [48].

### Variation of gene expansion and contraction

Chromosomal copy numbers were assessed as previously described in [48]. Briefly, chromosomal somy was determined using the read depth coverage per base normalised by the median genomic coverage for each library scaled as the haploid depth [48]. Gene expansions and contractions were identified by CNV estimations using the Control-FREEC package [50]. CNVs were identified in each isolate separately and regions of the genome that showed contractions or expansions exclusively in antimonial-treated isolates were assessed using *bedtools intersect* [51] and BEDOPS [52]. Gene Ontology Enrichment analyses in CNV regions were performed using TriTryp tools (https://tritrypdb.org/tritrypdb/app/) based on the orthologs between the *Leishmania tropica* and *Leishmania major* reference genomes.

### Functional impact of SNP changes

We examined the functional effects of published SNP data for 301,659 SNPs in this collection [48]. Previously, these had an average of 169,753 valid SNPs per sample with a standard deviation (SD) of 63,717 [48]. The chromosomal SNPs from the VCFs were annotated and their functional impact was inferred with SnpEff v5.0e [53] using the *L*. *tropica* LRC-L590 (MHOM/IL/1990/P283) reference genome assembly v2.0.2 and associated annotation from the GFF. We determined the nonsynonymous, synonymous and silent site changes across each of the 22 samples for each of their 36 chromosomes (22*36 = 792 regions). Nonsynonymous SNPs included; nonsense, missense, splice-related, stop codon gains/losses and start codon gains/losses. Selected mutations were visually checked with the Integrative Genomics Viewer (IGV) using the BAM files. The median Ti/Tv (transition/transversion) ratio per chromosome per sample was 2.18 (S.D. 0.16, range 1.62–2.87), suggesting that the broad patterns here aligned with our prior expectation of mutational constraint based on *L*. *donovani* [13].

To explore the relationship between heterozygosity changes and SNP functional effects, we computed the numbers of nonsynonymous polymorphisms (P_N_) and synonymous polymorphisms (P_S_) per chromosome per sample. The mean P_N_/P_S_ per chromosome (1.15) showed some variation across chromosomes (S.D. 0.98). Consequently, we assessed the rate of P_N_/P_S_ across the chromosomes and found no association with the Ti/Tv ratio (r^2^ = -0.0013, S1 Fig). For each chromosome per sample, we calculated the: heterozygosity based on the read-level patterns (mean±S.D. per chromosome 0.583±0.077), heterozygosity at nonsynonymous sites alone (0.591±0.092), and heterozygosity at synonymous sites (0.573±0.066). These heterozygosities were marginally higher than the disomic expectation of 0.5 because certain chromosomes were trisomic, tetrasomic or pentasomic, thus inflating average read-depth heterozygosity. We also examined known regions with unusual ancestry [48] or drug-related links [33], including the H-locus on chromosome 23 at bases 95,796–113,164. This data was parsed and visualised in R v4.1.0 (R Core Team 2021) with Dplyr v1.0.8 [54], Ggplot2 v3.3.5 [55], Ggrepel v0.9.1 [56], Grid v4.4.1, and Readxl x1.4.0 [57].

## Results

We examined the genomic variation in 22 *L*. *tropica* isolates to provide insights into the genetic basis of antimonial and non-antimonial treatment approaches applied and the varied treatment outcomes of the patients (Table 1).

### Variable heterozygosity per chromosome was associated with nonsynonymous changes

**Fig 1:**
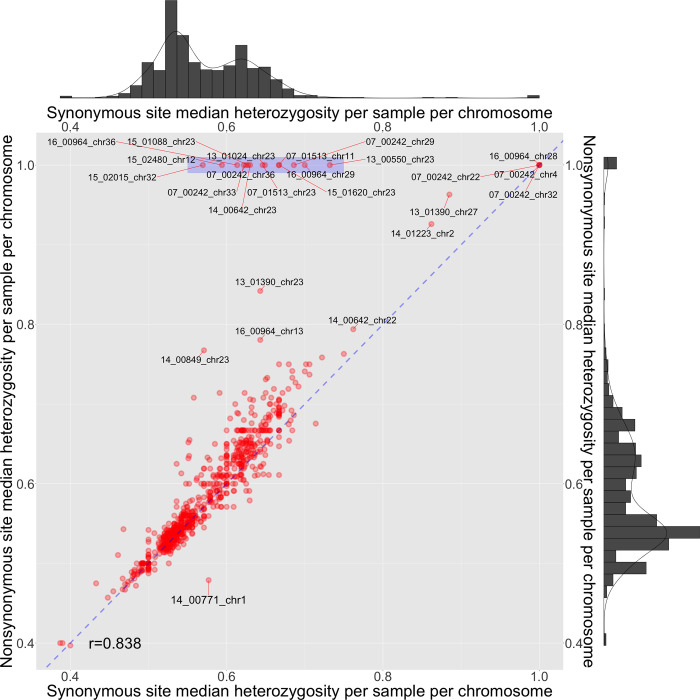
The association of the median nonsynonymous site heterozygosity (y-axis) with the median synonymous site heterozygosity (x-axis) for each of the 36 chromosomes in each of the 22 samples (n = 792, red points). The blue dashed line shows the hypothetical perfect correlation between the median nonsynonymous and synonymous site heterozygosities. Points at the top right indicate chromosomes with high nonsynonymous and synonymous site homozygosity. There was a positive association between nonsynonymous and synonymous site heterozygosities (adjusted r2 = 0.702). 14 chromosomes with median nonsynonymous site heterozygosities > 0.99 combined with median synonymous site heterozygosities < 0.75 (blue shaded area). Densigrams (top and right) show the frequency distributions such that the peaks correspond to disomic and trisomic read-depth allele frequencies. Chromosomes with extreme values are labeled.

There was considerable variation in heterozygosity between chromosomes and within chromosomes due to a combination of polysomic chromosomes, putative introgression events and recombination between homologous chromosomes [48]. We explored this further for all 36 chromosomes of each of the 22 samples using their heterozygosity rates at nonsynonymous sites and at synonymous ones. Although the nonsynonymous and synonymous heterozygosity rates were tightly correlated (r^2^ = 0.70), and unexpectedly 14 of the 792 chromosomes (1.8%) had median nonsynonymous site heterozygosities > 0.99 combined with median synonymous site heterozygosities < 0.75 (Fig 1, Table 2).

**Table 2:** 14 chromosomes had a median nonsynonymous site heterozygosities > 0.99 whose median synonymous site heterozygosities (“MedianHetS”) < 0.75 (Fig 1).

Sample	Country	Chr	PN/PS	Nonsyn	Syn	MedianHet	MedianHetS
07_00242	Iran	29	4.99	569	114	1.000	0.686
		33	2.00	663	332	0.750	0.622
		36	1.77	638	360	0.722	0.625
07_01513	Syria	11	2.18	286	131	0.821	0.667
		23^a^	1.78	346	194	0.765	0.646
13_00550	Syria	23^a^	2.20	216	98	1.000	0.732
13_01024	Syria	23^a^	2.12	349	165	0.817	0.649
14_00642	Syria	23^a^	2.20	319	145	0.750	0.630
15_01088	Syria	23^a^	2.09	451	216	0.857	0.628
15_01620	Syria	23^a^	2.07	396	191	0.789	0.667
15_02015	Syria	32	2.70	1830	678	1.000	0.570
15_02480	Afghanistan	12	2.76	677	245	1.000	0.594
16_00964	Iran	29	3.12	483	155	0.978	0.700
		36	1.72	434	252	0.571	0.614

For each of these 14, the median nonsynonymous site heterozygosity equals one and is not shown. The median site heterozygosities (“MedianHet”) rates above include non-coding SNPs. 07_00242 and 16_00964 both originated in Iran. 15_02480 was from Afghanistan, and the other seven samples shown here were from Syria. a = chromosome 23, with H locus. Chr = chromosome, nonsyn = nonsynonymous, syn = synonymous

Moreover, this homozygosity was correlated with much higher P_N_/P_S_ ratios for a small minority of samples’ chromosomes, particularly 07_00242, unlike the majority with normal heterozygosity corresponding to the somy level and with P_N_/P_S_ approximating one (S2 Fig). Most chromosomes did not have this pattern, it was clear that some had sharp changes in P_N_/P_S_ ratios, heterozygosity and nonsynonymous SNP density (S3 Fig).

### High rates of nonsynonymous change at the H-locus on chromosome 23

**Fig 2:**
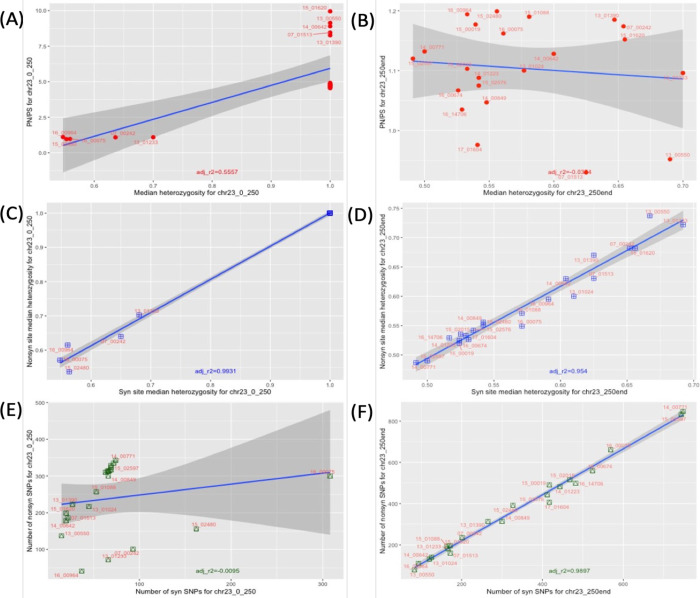
Contrasting rates of heterozygosity and P_N_/P_S_ ratios for chromosome 23’s 5’ (<250 Kb, A/C/E) and 3’ (>250 Kb, B/D/F) ends. P_N_/P_S_ ratios were high for the 17 samples with homozygosity (red circles) but not the five heterozygous samples at the 5’ end (A), whereas the 3’ end had no such disparity (B). The median nonsynonymous and synonymous heterozygosity per SNP was equal to one for these 17 homozygous samples (blue squares) at the 5’ end (C), contrasting with the trend at the 3’ end (D). The P_N_/P_S_ rates were more correlated for the 17 homozygous samples (green squares) than the five heterozygous ones at the 5’ end (E), unlike the 3’ one that showed a more typical pattern (F). For each plot, the line of best fit (blue), its 95% confidence interval (grey area) and the associated r^2^ value are shown.

Previously, the 5’ end (0–250 Kb) of chromosome 23 was found to contain an unusually long run of homozygosity in most samples in this collection (17 out of 22) [48], and here five samples had relatively elevated nonsynonymous site heterozygosity at chromosome 23 (S2 Table). Across this whole chromosome, these 17 highly homozygous isolates had a significantly higher median P_N_/P_S_ compared to the five samples with normal heterozygosity (1.67 vs 1.12, Wilcoxon signed rank p = 0.011, S2 Table), and the median P_N_/P_S_ per sample across all 36 chromosomes (1.04). Furthermore, these 17 samples had P_N_/P_S_ > 4 at the 5’ end (0–250 Kb) of chromosome 23, unlike the heterozygous five samples that had P_N_/P_S_ < 1.2, and the P_N_/P_S_ ratios were positively correlated within each group, but at different levels (S4 Fig). In contrast, the 3’ end (>250 Kb) of this chromosome showed no such differences between these groups nor their P_N_/P_S_ ratios (Fig 2), indicating that the jump in nonsynonymous SNPs in the 17 samples 3’ ends of chromosome 23 were linked to their long runs of homozygosity.

The 5’ end of this chromosome showed a consistent pattern of nonsynonymous change associated with different ancestry, including the well-characterised drug resistance linked H-locus at bases 95,796 to 113,164. The length (250 Kb) of this difference and contrast with the 5’ end suggested that the considerable changes observed were unlikely explained by relaxed selective constraint alone. The 17 homozygous samples came from patients originally from Syria, and the five heterozygous ones were from patients originally from Iran and Afghanistan (07_00242, 13_01233, 15_02480, 16_00075 and 16_00964) (37). 07_00242 was the only sample among the five that had received treatment prior to sampling but showed no alteration in P_N_/P_S_ rate compared to the others. Several SNPs in isolates from Afghanistan and Iran (07_00242, 13_01233, 15_02480, 16_00075) were at the H-locus, which has been associated with treatment failure and drug resistance (S3 Table).

In a prior study, we found discrete changes in heterozygosity and ancestry at chromosome 36 that were linked to recombination during parasexual mating [48]. Although homozygous regions at the 5’ end (<1.30 Mb) of this chromosome were not noticeably associated with elevated P_N_/P_S_ rates (S5 Fig), 07_00242 and 16_00964 had elevated homozygosity at 1.30–1.78 Mb, P_N_/P_S_ > 4 and a higher relative rate of nonsynonymous SNPs (S5 Fig) that corresponded to a segment of homozygous ancestry dissimilar to the reference genome [48]. This ancestry and homozygosity-nonsynonymous SNP association was evident in 14_01223 at 1.60–1.78 Mb (S5 Fig), in line with the previous work [48]. 16_00075 had a similar effect to a smaller degree, and for 14_00642 the association was not apparent (S5 Fig). As per this previous study [42], 07_00242, 16_00964 and 14_01223 had high homozygosity similar to the reference genome at the 3’ end of this chromosome 36 (>1.80 Mb) that retained the homozygosity-nonsynonymous SNP correlation, which was not found in the samples with typical heterozygosity, 16_00075 and for 14_00642 (S5 Fig).

Previously, we hypothesised that chromosome 10 from 4_00642 was monosomic at 20–250 Kb and approximately trisomic at >250 Kb (37). Here, 14_00642 had a median heterozygosity of 1.0, a high P_N_/P_S_ rate of 4.3, and a higher median nonsynonymous than synonymous heterozygosity (0.99 v 0.74) at the monosomic region (S6 Fig). In contrast, the trisomic region >250 Kb with the median heterozygosity of 0.67, matching the expected value for a trisomic chromosome, a normal P_N_/P_S_ (1.1), and no difference between the nonsynonymous and synonymous median heterozygosities (S6 Fig). This supported the monosomy-trisomy switch and linked it to amino acid changes at the region at 20–250 Kb.

Our previous work highlighted a long run of homozygosity associated with rare ancestry at chromosome 29 >440 Kb and > 490 Kb for two samples from Iran, 16_00964 and 07_00242, respectively [42]. In contrast to the normal pattern of heterozygosity found at the 5’ end of this chromosome for these two samples and the other 20, 07_00242 and 16_00964 had high P_N_/P_S_ rates (4.99 and 3.12) and elevated nonsynonymous site homozygosity (S7 Fig).

Further, we analyzed the effects of SNPs in the 14 chromosomes with higher nonsynonymous mutation rates (Table 2). 0.06% of SNPs in all 792 chromosomes generated stop codons, whereas 0.29% of SNPs generated stop codons in genes encoded in these 14 chromosomes. Most of the affected genes encode for hypothetical proteins and a minority are associated with redox metabolism (S4 Table). To assess the contribution of these nonsynonymous mutations throughout these chromosomes, we performed a gene ontology analysis of all the genes with nonsynonymous SNPs (excluding those generating stop codons) in the 14 chromosomes (S5 Table). Interestingly, nonsynonymous mutations were observed in genes related to response to a stimulus such as ABC transporters, multidrug resistance protein A, and DNA damage repair, suggesting these SNPs could contribute to the variability of proteins associated with pathogenesis.

No correlation between P_N_/P_S_ and antimonial treatment after sampling, group B, were observed.

### Gene expansion in isolates exposed to antimonial

To gain insight on the H locus’ SNP variation on chromosome 23, in isolates from Syria, a CNV analysis was performed to see if there was variation in gene dosage as well. The isolates were grouped into two based on exposure to antimonial treatment: 9 previously treated with antimony in group A, and 12 with no previous exposure to antimony in group B (Table 1).

**Fig 3:**
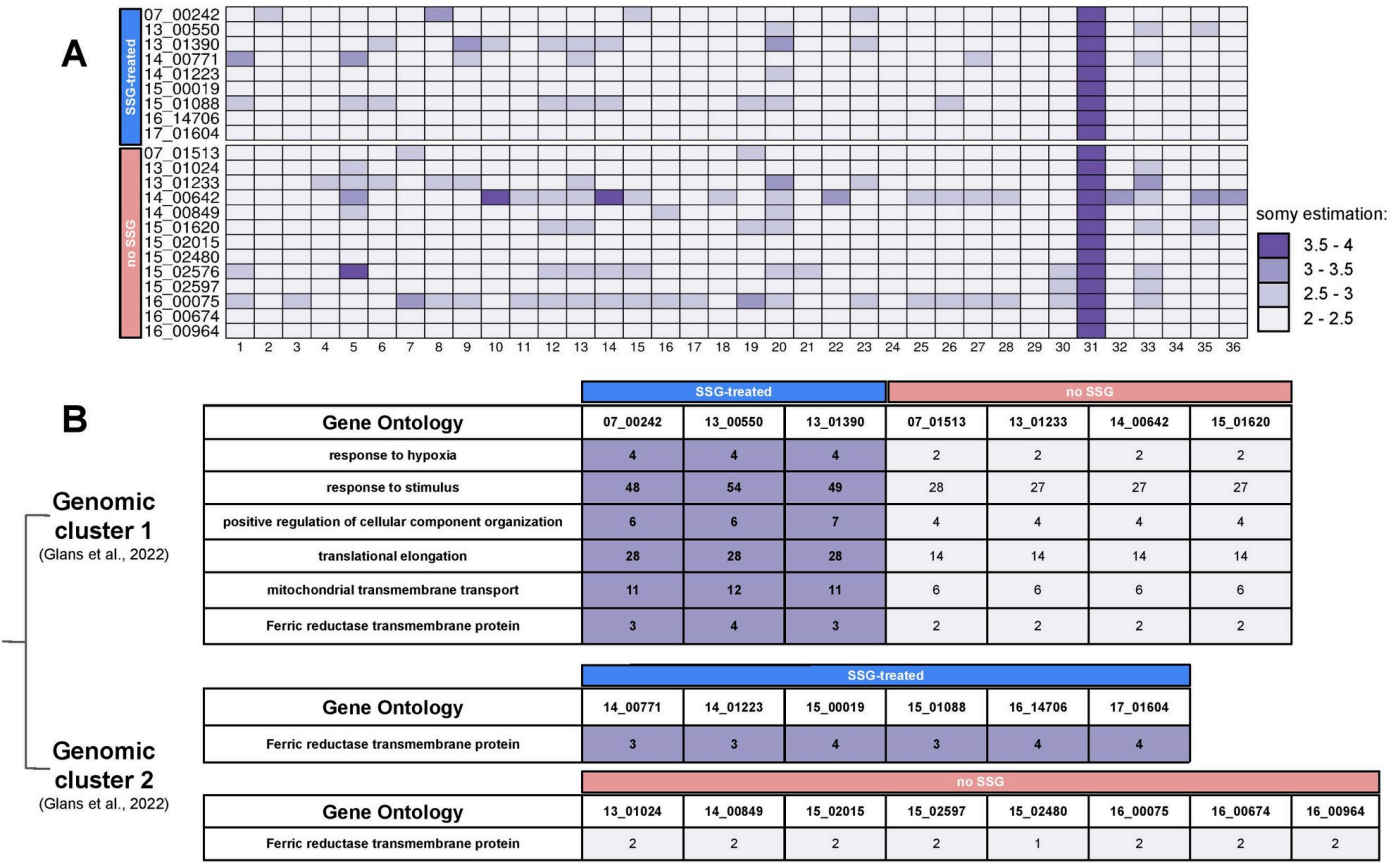
Chromosomal and genomic copy number variations in *L*. *tropica* clinical isolates. A. Heat Map showing aneuploidies. B. Expanded regions plot.

Chromosome somy was assessed as in [48] and no patterns were associated with antimonial treatment (Fig 3A).

We evaluated CNV in each isolate separately and compared the regions of the genome that showed contractions or expansions in exclusively antimonial-treated isolates. The gene LmjF.30.1610, coding for a ferric reductase transmembrane protein, was the only expanded coding region in all isolates previously treated with antimonial, group A. No other patterns of genome contraction were common to all isolates.

Due to the high degree of genomic diversity in the isolates, we also separated the samples into two clusters according to the two genetic groups, “reference” and “non-reference” (Table 1) [48]. Here, we could identify 204 genes (S6 Table) that were expanded exclusively in previously antimonial treated isolates from cluster “reference” (07_00242, 13_00550, 13_01390). By performing gene ontology and gene enrichment analysis of these expanded genes, we identified five main functional categories that had previously all been linked to drug resistance to antimonial: i) translational elongation; ii) mitochondrial transmembrane transport; iii) positive regulation of cellular component organisation; iv) response to stimulus; and v) response to hypoxia (Fig 3B). A list of genes expanded for each gene ontology category is available in the S7 Table. Regarding genes involved in translational elongation, 7 copies of the elongation factor 1-alpha gene were on chromosome 17 (S7 Table). Regarding mitochondrial transmembrane transport, a gene encoding a mitochondrial pyruvate carrier was expanded on chromosome 1 and another encoding a mitochondrial import receptor subunit ATOM69 was expanded on chromosome 28 (S7 Table). In the positive regulation of cellular component organisation category, a gene encoding eukaryotic initiation factor 5A was expanded on chromosome 25 (S7 Table). Finally, in response to hypoxia, a gene encoding oxygen-sensing adenylate cyclase was expanded on chromosome 28 (S7 Table). In cluster “non-reference” and previous antimonial exposure (Table 1) (14_00771, 14_01223, 15_00019, 15_01088, 16_14706 and 17_01604), only gene LmjF.30.1610 encoding a putative ferric reductase was expanded in all isolates previously treated with antimonial.

No patterns of CNV were identified in the H locus, chromosome 23, MAPK1, chromosome 36, or APQ1, chromosome 31, with or without previous antimonial exposure. In group B, the four isolates, receiving antimonial treatment after sampling, were too diverse to observe any correlation between variation in CNV and antimonial treatment outcome after sampling.

## Discussion

Our results show a complex genetic picture of the 22 isolates of *L*. *tropica*. We found numerous nonsynonymous SNPs driven by changes in ancestry and somy, as well as extensive variation in gene copy numbers.

We observed a higher frequency of nonsynonymous SNPs in the genomes of isolates originating from Afghanistan/Iran (07_00242, 13_01233, 15_02480, 16_00075 and 16_00964) compared to those from Syria. No correlation with the previously described population structure [48] or previous exposure to antimony was observed (Table 1). A difference in median nonsynonymous site heterozygosity relative to the median synonymous site heterozygosity correlated to geographic variation between Syria and Afghanistan/Iran was detected. The *L*. *tropica* genome has extensive plasticity, similar to other *Leishmania* species [48]. Frequent genetic exchange driven by recombination and aneuploidy suggest the occurrence of hybrid formation and rapid diversification in the parasite [48]. In our study, more frequent heterozygosity, genetic exchange, recombination and aneuploidy were present in isolates from Syria. Higher homozygosity and less frequent genetic exchange were evident in isolates from Afghanistan and Iran.

In this context of a dynamical *L*. *tropica* genome, we found that heterozygous isolates from Syria [48] had more synonymous SNPs, unlike the homozygous isolates from Afghanistan and Iran which had more nonsynonymous SNPs. A higher P_N_/P_S_ rate with high homozygosity and relatively more nonsynonymous SNPs were seen among isolates from Syria on chromosome 23 at the H locus compared to isolates from Afghanistan/Iran. This indicated that genetic diversity is driven by geographic separation at the H locus. The chance of a stop codon on the 14 chromosomes with increased nonsynonymous SNPs was slightly higher than for all 792 chromosomes (0.29% vs 0.06%). Most of the affected genes encode for proteins without orthologs at *L*. *major* genome (42%), hypothetical proteins (27%), and only two genes associated with redox metabolism (*Tryparedoxin* and *Ubiquinone biosynthesis protein*). Even though oxidative stress plays a crucial role in pathogenesis, only two genes involved in this process was affected by the stop codons. In most cases, the nonsynonymous SNP was a missense change with an unknown effect to the protein expressed. Interestingly, nonsynonymous mutations were observed at genes related to responses to stimuli such as ABC transporters, multidrug resistance protein A, and DNA damage repair, suggesting these SNPs could contribute to the variability of proteins associated with pathogenesis. These results are in line with a previous finding of adaptation via SNPs with no sizable changes in gene expression [13].

Rapid generation, mechanistic independence and convergent evolutionary patterns of genetic variants associated with antimony pressure have previously occurred in different geographically dispersed *L*. *donovani* [20, 30, 33] and *L*. *tropica* populations [58]. These local variants evolved prior to antimony exposure [30, 33] and may be adaptations to local environmental changes [33, 59]. However, in our study the high frequency of homozygosity at chromosome 23´s 5’ end (< 250 Kb) in the 17 isolates from Syria were not correlated with either gene dosage or previous antimony exposure. The variation in P_N_/P_S_ at chromosome 23 in isolates from Syria (S6 Table) may be an effect of recombination and hybridizations in this lineage as previously described [48].

A gain of function through the amplification of genes and CNV as described in other *Leishmania* spp. [12, 31, 33, 60] has often correlated with environmental changes and drug pressure [19, 33, 60]. In our isolates, several genes with functions important for parasite survival were affected. These functions included: coping mechanisms to suppress and survive oxidative stress, DNA repair system after oxidative damage, the trypanothione pathway to reduce the oxidative stress, cell wall stability, iron and heme metabolism and parasite viability and virulence. Several genes in these functional categories were amplified, possibly as a response to drug pressure (Fig 3, S7 Table). Differences in amplified genes´ functional categories were observed between the two geographical regions. In isolates from Syria, these genes were associated with DNA replication, whereas in Afghanistan/Iran genes driving apoptosis had lower copy numbers. The use of gene dosage as an adaptive mechanism by *L*. *tropica* and its correlation with clinical outcome when exposed to drugs were suggested previously [18]. Similar events have been observed in antimony-resistant *L*. *infantum* [21] and methotrexate-resistance *L*. *donovani* and *L*. *tropica* [39, 40].

Ferric iron reductase is a bifunctional protein that generates both ferrous iron and hydrogen peroxide (H2O2) [61]. Here, the gene encoding this enzyme was amplified in all isolates exposed to antimony. H2O2 is needed to acquire heme from the host and is involved in both oxidative metabolism and antioxidant defense, and is used as a signal molecule [61]. If the parasite lacks ferric iron reductase, it cannot grow intracellularly nor establish an infection in the host [62].

Most genetic data related to drug resistance has previously been produced for *L*. *donovani* and *L*. *infantum* in the clinical context of VL [20, 33], and for *L*. *braziliensis* and *L*. *panamensis* [31]. *L*. *tropica* causes CL and leishmaniasis recidivans and is present in different geographic areas. It uses diverse sand fly species as vectors and different transmission routes in separate geographic areas, which could result in novel genetic mechanisms not previously observed in other *Leishmania* spp. Nonetheless, the limited number of isolates from different geographic areas may affect the genomic heterogeneity seen between isolates from Iran/Afghanistan and Syria.

Long-term culturing and environmental conditions *in vitro* affect *Leishmania* genome diversity [20, 63, 64]. Our 22 isolates were cultured while diagnosed, stored at -156 C and then re-cultured, in axenic culture *in vitro*, as promastigotes for two to six weeks before DNA was sequenced. This may have affected some genetic variation.

The number of isolates is a limitation: consequently, it is pertinent to interpret the results with caution. We were unable to evaluate antimonial treatment after sampling in group B due to the limited number of samples. Information on treatment dose and duration prior to sampling and confirmation of the susceptibility to the antimony of the isolates could have supported the interpretation of the results.

*L*. *tropica* has several genetic mechanisms to adapt to environmental changes and drug pressure, such as nonsynonymous SNP changes and extensive variation in CNV. Previous work has also emphasised extensive heterogeneity among somy levels in *L*. *tropica* [18, 19] and related *Leishmania* species [13, 20], features that were not clearly associated with antimonial treatment here. Some mechanisms may not be driven by historical antimonial usage but may have evolved to support *L*. *tropica* durability in adverse contexts, such as niches with higher rates of heavy metals. Moreover, the environmental challenges of the different life cycle stages and in different zoonotic hosts may elicit a capability for rapid genetic responses. The elevated frequency of H locus SNPs in isolates from Syria may be an indication of adaptation to antimonial.

Further research is needed to better understand the correlation between genetic diversity and different genetic mechanisms used for *Leishmania* to survive treatment and adapt to environmental changes. By examining different components of genome-wide diversity in *L*. *tropica*, we found multiple independent genetic mechanisms of parasite survival under drug pressure. These results provide candidates for future functional studies of *L*. *tropica* drug adaptation and evolution.

## Supporting information

S1 Fig There was no evidence of an association between the P_N_/P_S_ values (y-axis) and the Ti/Tv (transition/transversion) rates (x-axis) per sample per chromosome (r^2^ = -0.0013). The inset shows the same data restricted to P_N_/P_S_ < 4. Densigrams show the distributions of the Ti/Tv (top) and PN/PS (right) per chromosomes. The Ti/Tv ratios per sample per chromosome showed a narrow range of variation, suggesting no chromosome-wide evidence of a relaxed selective constraint.
(TIF)

S2 Fig For all 36 chromosomes for each of the 22 samples (n = 792 items), there was a positive association of P_N_/P_S_ with homozygosity (r^2^ = 0.25), particularly for certain 07_00242 chromosomes. The inset of values with P_N_/P_S_ < 4, showing the details for other samples’ chromosomes, whose P_N_/P_S_ tended to coincide with more homozygosity. The P_N_/P_S_ is the number of nonsynonymous SNPs divided by the number of synonymous SNPs.
(TIF)

S3 Fig For each chromosome, the association between (A) P_N_/P_S_ rates (y-axis) with heterozygosity (x-axis) (each sample is a red dot); (B) the synonymous site heterozygosity (x-axis) versus the nonsynonymous site heterozygosity (y-axis) (each sample is a blue square); and (C) the numbers of synonymous SNPs (x-axis) versus the numbers of nonsynonymous SNPs (y-axis). The blue line per plot is a linear model of the best fit between the two variables across the samples, with the 95% confidence interval shown by the grey area. The adjusted r^2^ value for this model is shown on each plot.
(PDF)

S4 Fig For chromosome 23, 07_00242, 13_01233, 15_02480, 16_00075 and 16_00964 had substantially lower P_N_/P_S_ rates across a range of heterozygosity levels (A). This was linked to reduced nonsynonymous relative to synonymous site heterozygosity (B), and a dearth of nonsynonymous SNPs (C). All five isolates came from patients originally from Iran or Afghanistan, and previously had a substantial long run of homozygosity at <250 Kb on this chromosome. The average P_N_/P_S_ rate across all 22 samples for this chromosome was higher than the other chromosome’s (1.50 vs 1.06+-0.14, 99.9^th^ percentile).
(TIF)

S5 Fig For chromosome 36’s region <1.28 Mb, (A) the P_N_/P_S_ ratios, heterozygosity rates, (B) nonsynonymous and synonymous site heterozygosities, and (C) numbers of nonsynonymous and synonymous SNPs were as expected. This contrasted with the adjacent region at 1.30–1.65 Mb, where 07_00242’s and 16_00964’s (16_00075’s to a lesser extent) high P_N_/P_S_ rates were associated with less heterozygosity (D), 07_00242 had a higher nonsynonymous relative to synonymous site heterozygosity (E), and these high P_N_/P_S_ rates were due to a higher number of nonsynonymous SNPs (F). At 1.60–1.78 Mb, 07_00242’s, 16_00964’s, 14_01223’s and 16_00075’s high P_N_/P_S_ rates were again associated with less heterozygosity (G), but not a higher nonsynonymous relative to synonymous site heterozygosity (H), nor were the high P_N_/P_S_ rates were due to a higher number of nonsynonymous SNPs (I). At >1.8 Mb, 07_00242’s, 14_01223’s and 16_00964’s high P_N_/P_S_ rates were associated with less heterozygosity (J), not a higher nonsynonymous relative to synonymous site heterozygosity (K), and not to higher numbers of nonsynonymous SNPs (L).
(TIF)

S6 Fig At chromosome 10, 14_00642 had a (A) high P_N_/P_S_ associated with less heterozygosity at the 5’ end (20–270 Kb), unlike the 3’ end (>270 Kb) that was like the other samples. (C) 14_00642 had a higher nonsynonymous relative to synonymous site heterozygosity at the 5’ end too, but (D) not at the 3’ end. Similarly, (E) 14_00642 had more nonsynonymous SNPs at the 5’ end, unlike (F) at the 3’ end.
(TIF)

S7 Fig For chromosome 29’s (A) 5’ end at 0–440 Kb, there was no large associations of the heterozygosity with PN/PS, (B) unlike the 3’ end (>490 Kb) where 07_00242’s and 16_00964’s high PN/PS rates were associated with less heterozygosity. (C) The 5’ end had neutral nonsynonymous and synonymous site heterozygosities, whereas (D) 07_00242’s and 16_00964’s rates were high for both. (E) The 5’ end of these samples had equivalent numbers of nonsynonymous SNPs and synonymous SNPs, (F) but the 3’ end did not–both had an excess of nonsynonymous SNPs. Both came from patients originally from Iran.
(TIF)

S1 Table Published gene variations in the H locus, on chromosome 23, correlated to drug resistance. (DOCX)

S2 Table Genes expanded exclusively in antimonial-treated isolates from cluster 1. (DOCX)

S3 Table List of genes expanded per gene ontology category. (DOCX)

S4 Table Gene ontology analysis of genes with gained stop codons in the 14 target chromosomes. Bgd count: Number of genes with this term in the genome; Result count: Number of genes with this term in this analysis; Pct of bgd: Of the genes in the background with this term, the percent that is present in the result. Fold enrichment: The percent of genes with this term in this analysis divided by the percent of genes with this term in the genome.
(DOCX)

S5 Table Gene ontology analysis of genes with nonsynonymous SNPs in the 14 target chromosomes. Bgd count: Number of genes with this term in the genome; Result count: Number of genes with this term in this analysis; Pct of bgd: Of the genes in the background with this term, the percent that is present in the result. Fold enrichment: The percent of genes with this term in this analysis divided by the percent of genes with this term in the genome.
(DOCX)

S6 Table Gene ontology analysis of exclusively expanded genes in isolated previously treated with anitmonial. Bgd count: Number of genes with this term in the genome; Result count: Number of genes with this term in this analysis; Pct of bgd: Of the genes in the background with this term, the percent that is present in the result. Fold enrichment: The percent of genes with this term in this analysis divided by the percent of genes with this term in the genome.
(DOCX)

S7 Table List of genes expanded for the five main categories previously linked to drug resistance to antimonial drugs. (DOCX)

S1 Questionnaire Inclusivity in global research. (DOCX)

## Abbreviations

AQP1: aquaglyceroporin 1
MAPK-1: mitogen- activated protein kinase
VL: visceral leishmaniasis
