# Supplementary figures and images for: Genetic coping mechanisms observed in Leishmania tropica, from the Middle East region, enhance the survival of the parasite after drug exposure
Source: PLoS One. 2024 Dec 3;19(12):e0310821. doi: 10.1371/journal.pone.0310821 (PMC11614225; doi:10.1371/journal.pone.0310821)

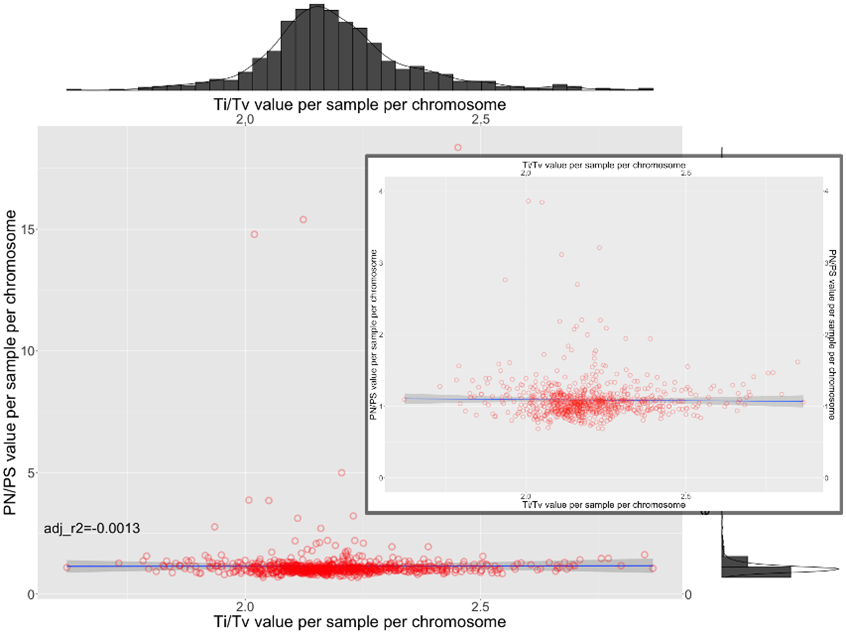

Supplement: S1 Fig — The inset shows the same data restricted to PN/PS < 4. Densigrams show the distributions of the Ti/Tv (top) and PN/PS (right) per chromosomes. The Ti/Tv ratios per sample per chromosome showed a narrow range of variation, suggesting no chromosome-wide evidence of a relaxed selective constraint. (TIF) [file pone.0310821.s001.tif]

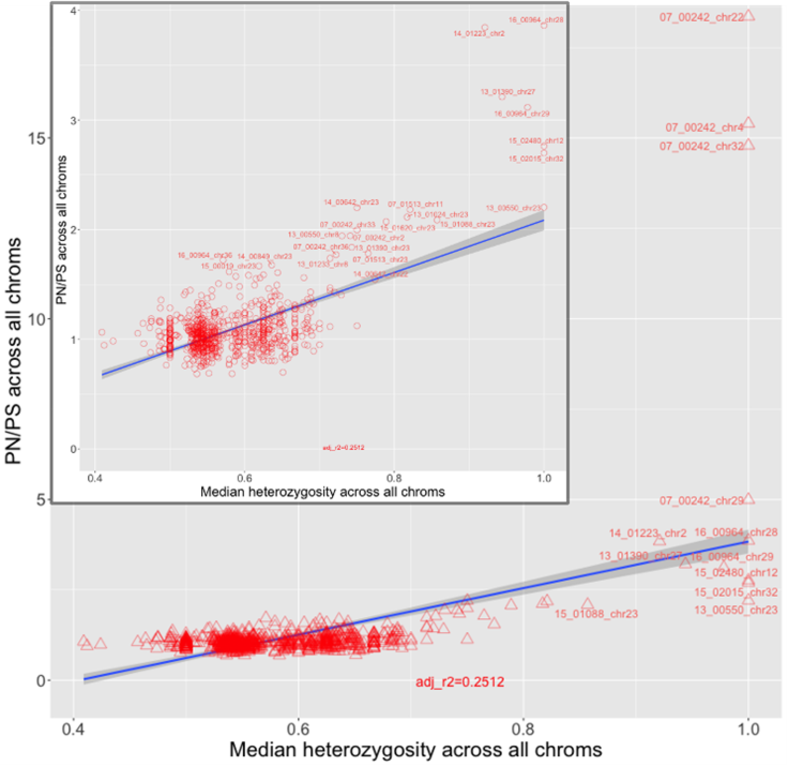

Supplement: S2 Fig — The inset of values with PN/PS < 4, showing the details for other samples’ chromosomes, whose PN/PS tended to coincide with more homozygosity. The PN/PS is the number of nonsynonymous SNPs divided by the number of synonymous SNPs. (TIF) [file pone.0310821.s002.tif]

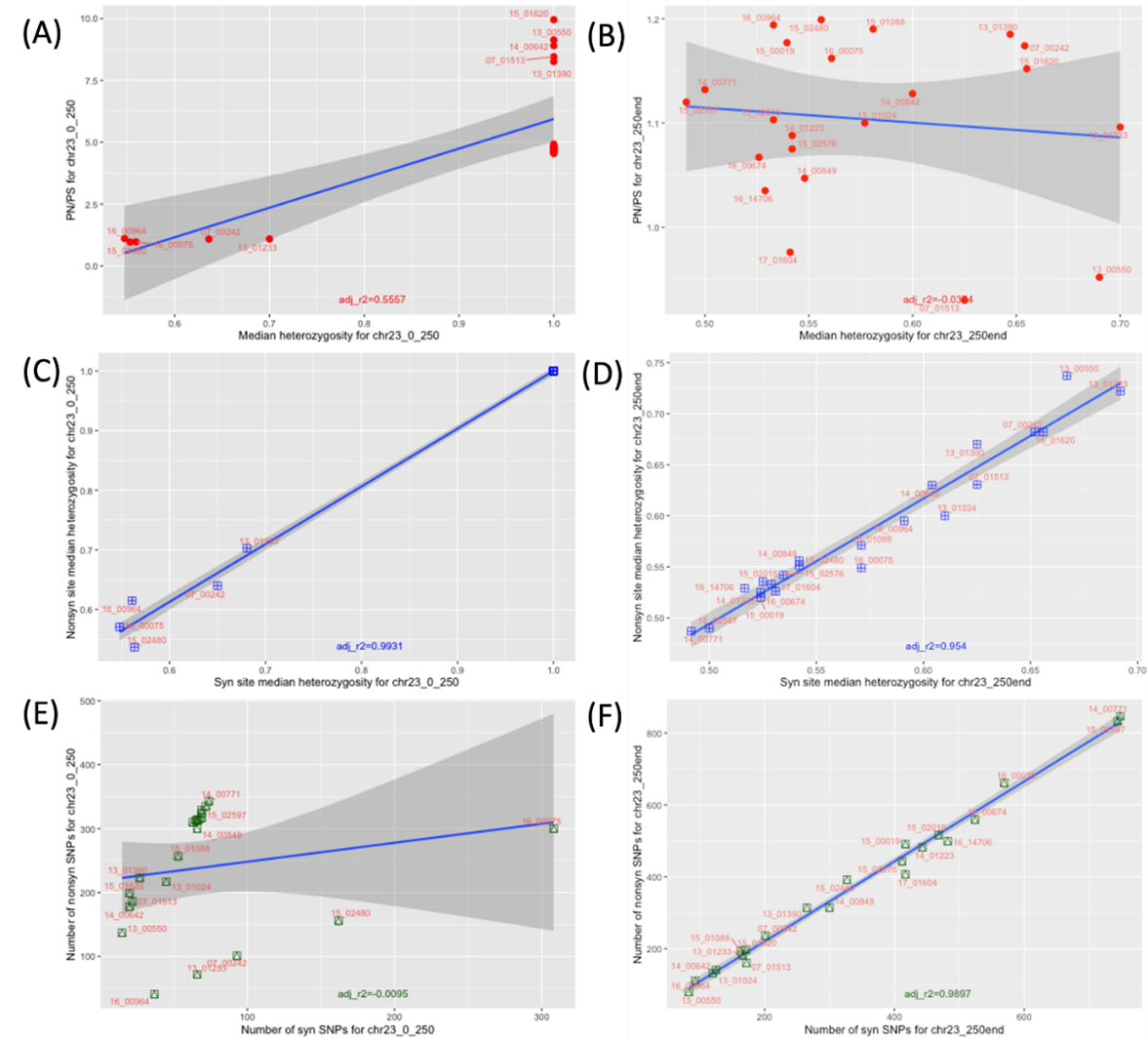

Supplement: S4 Fig — For chromosome 23, 07_00242, 13_01233, 15_02480, 16_00075 and 16_00964 had substantially lower PN/PS rates across a range of heterozygosity levels (A). This was linked to reduced nonsynonymous relative to synonymous site heterozygosity (B), and a dearth of nonsynonymous SNPs (C). All five isolates came from patients originally from Iran or Afghanistan, and previously had a substantial long run of homozygosity at <250 Kb on this chromosome. The average PN/PS rate across all 22 samples for this chromosome was higher than the other chromosome’s (1.50 vs 1.06+-0.14, 99.9th percentile). (TIF) [file pone.0310821.s004.tif]

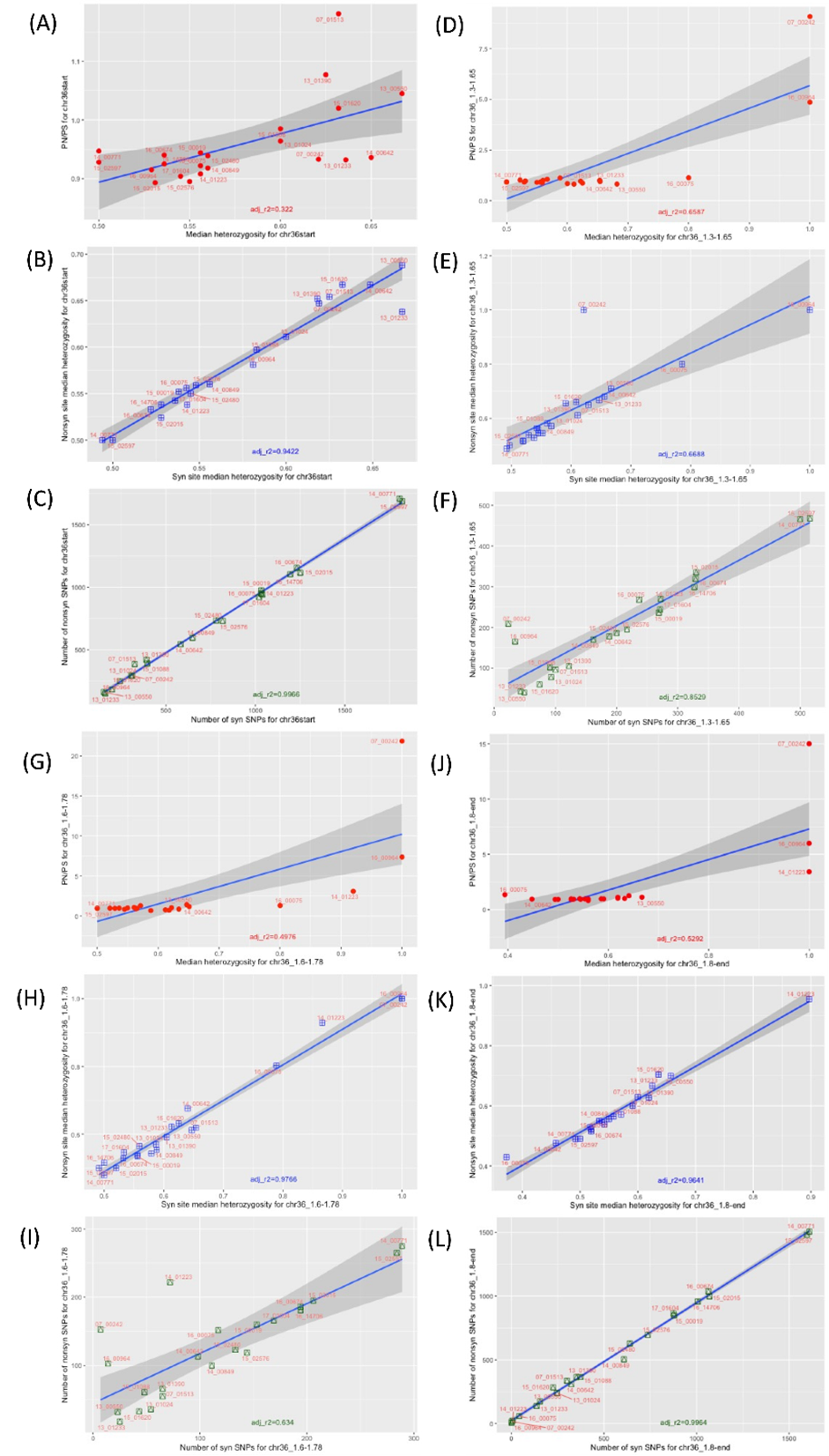

Supplement: S5 Fig — For chromosome 36’s region <1.28 Mb, (A) the PN/PS ratios, heterozygosity rates, (B) nonsynonymous and synonymous site heterozygosities, and (C) numbers of nonsynonymous and synonymous SNPs were as expected. This contrasted with the adjacent region at 1.30–1.65 Mb, where 07_00242’s and 16_00964’s (16_00075’s to a lesser extent) high PN/PS rates were associated with less heterozygosity (D), 07_00242 had a higher nonsynonymous relative to synonymous site heterozygosity (E), and these high PN/PS rates were due to a higher number of nonsynonymous SNPs (F). At 1.60–1.78 Mb, 07_00242’s, 16_00964’s, 14_01223’s and 16_00075’s high PN/PS rates were again associated with less heterozygosity (G), but not a higher nonsynonymous relative to synonymous site heterozygosity (H), nor were the high PN/PS rates were due to a higher number of nonsynonymous SNPs (I). At >1.8 Mb, 07_00242’s, 14_01223’s and 16_00964’s high PN/PS rates were associated with less heterozygosity (J), not a higher nonsynonymous relative to synonymous site heterozygosity (K), and not to higher numbers of nonsynonymous SNPs (L). (TIF) [file pone.0310821.s005.tif]

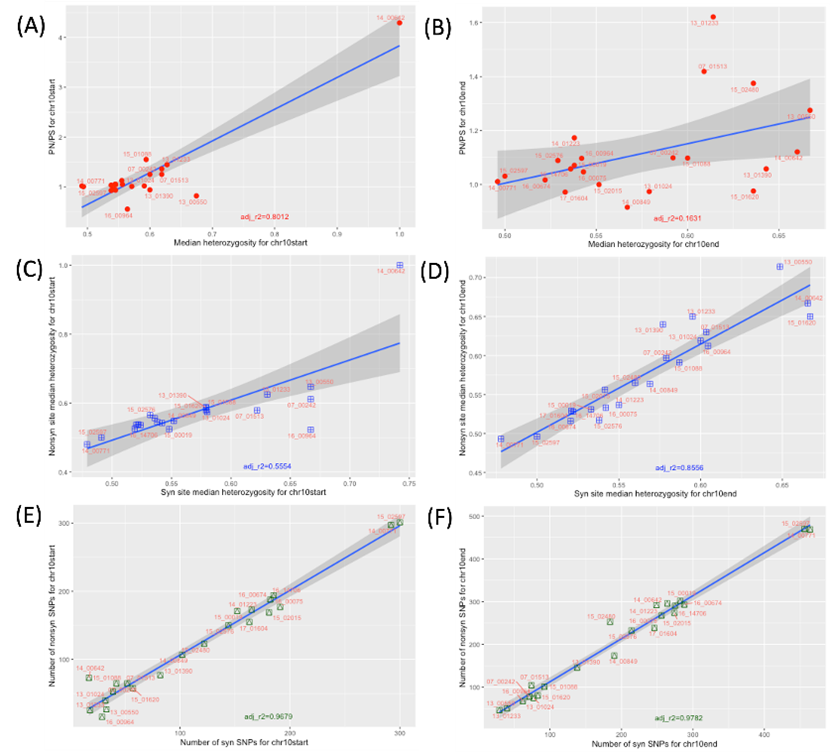

Supplement: S6 Fig — At chromosome 10, 14_00642 had a (A) high PN/PS associated with less heterozygosity at the 5’ end (20–270 Kb), unlike the 3’ end (>270 Kb) that was like the other samples. (C) 14_00642 had a higher nonsynonymous relative to synonymous site heterozygosity at the 5’ end too, but (D) not at the 3’ end. Similarly, (E) 14_00642 had more nonsynonymous SNPs at the 5’ end, unlike (F) at the 3’ end. (TIF) [file pone.0310821.s006.tif]

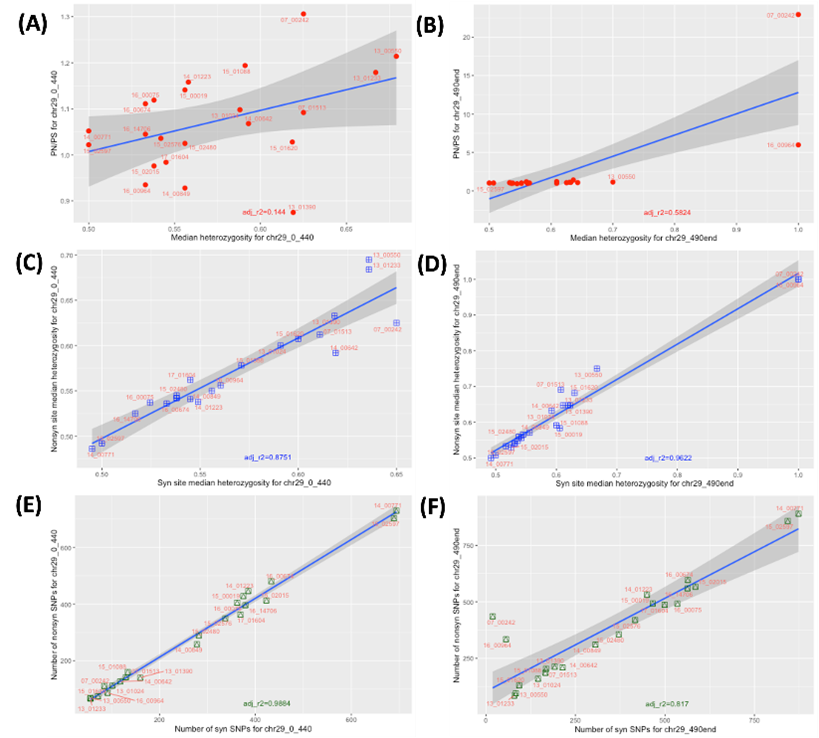

Supplement: S7 Fig — For chromosome 29’s (A) 5’ end at 0–440 Kb, there was no large associations of the heterozygosity with PN/PS, (B) unlike the 3’ end (>490 Kb) where 07_00242’s and 16_00964’s high PN/PS rates were associated with less heterozygosity. (C) The 5’ end had neutral nonsynonymous and synonymous site heterozygosities, whereas (D) 07_00242’s and 16_00964’s rates were high for both. (E) The 5’ end of these samples had equivalent numbers of nonsynonymous SNPs and synonymous SNPs, (F) but the 3’ end did not–both had an excess of nonsynonymous SNPs. Both came from patients originally from Iran. (TIF) [file pone.0310821.s007.tif]
