# Supplementary material for: Genetic coping mechanisms observed in Leishmania tropica, from the Middle East region, enhance the survival of the parasite after drug exposure
Source: PLoS One. 2024 Dec 3;19(12):e0310821. doi: 10.1371/journal.pone.0310821 (PMC11614225; doi:10.1371/journal.pone.0310821)

S3 Figure.

Figure 1. For chromosome 1, there was no clear association of PN/PS with heterozygosity (A), and although there were differences in nonsynonymous relative to synonymous site heterozygosity these were quite mixed (B), as was the relative number of nonsynonymous SNPs (C).

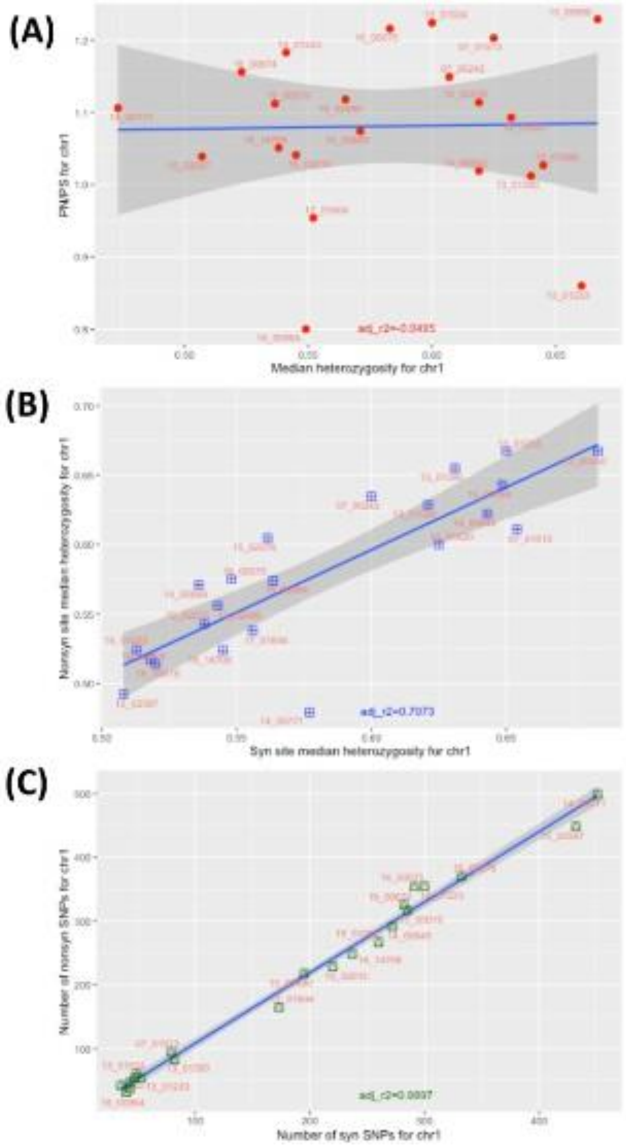

Figure 2. For chromosome 2, 14_01223 had a high PN/PS of 3.85 that was associated with more homozygosity (A), but not a difference in nonsynonymous relative to synonymous site heterozygosity (B), whereas the high PN/PS was due to a high number of nonsynonymous SNPs (323 vs 84 for synonymous SNPs) (C). 14_01223 and 17_01604 were from the same patient originally from Syria but were genetically distinct. 14_01223 was initially treated with intravenous SSG for nine days that failed before a cure with LA treatment (Table 1). Here, 14_01223 had 54 mutated genes with 318 nonsynonymous and 82 synonymous SNPs. For comparison, 15_02015 had a more typical PN/PS with 55 mutated genes possessing 292 nonsynonymous and 212 synonymous SNPs.

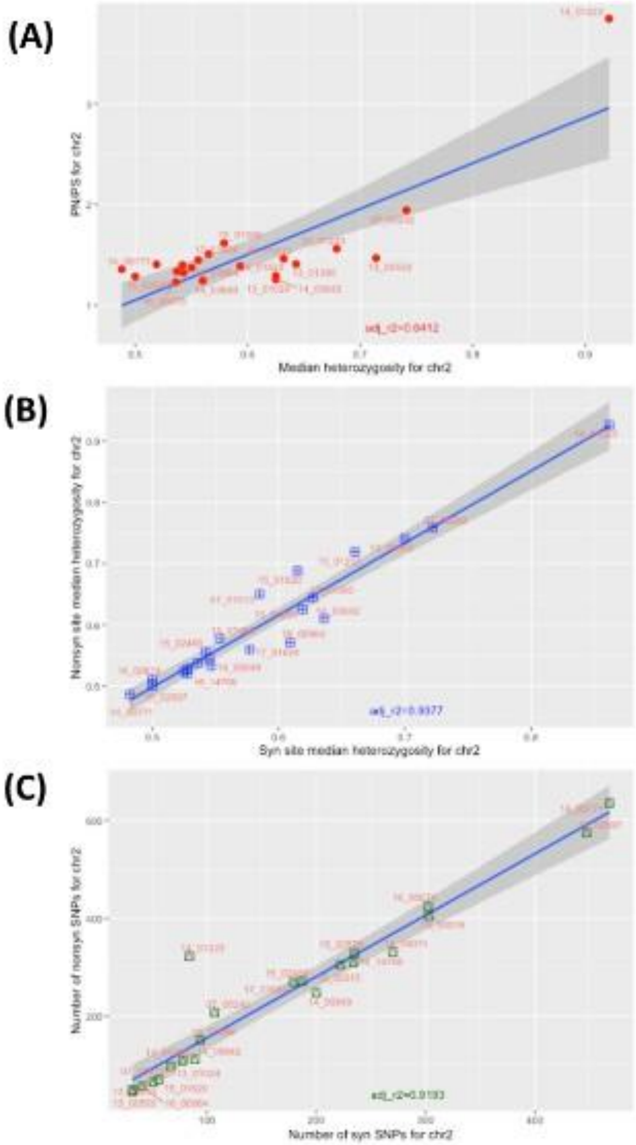

Figure 3. For chromosome 3, 15_01620's high PN/PS rate was associated with less heterozygosity (A), but not a higher nonsynonymous relative to synonymous site heterozygosity (B), nor a higher number of nonsynonymous SNPs (C).

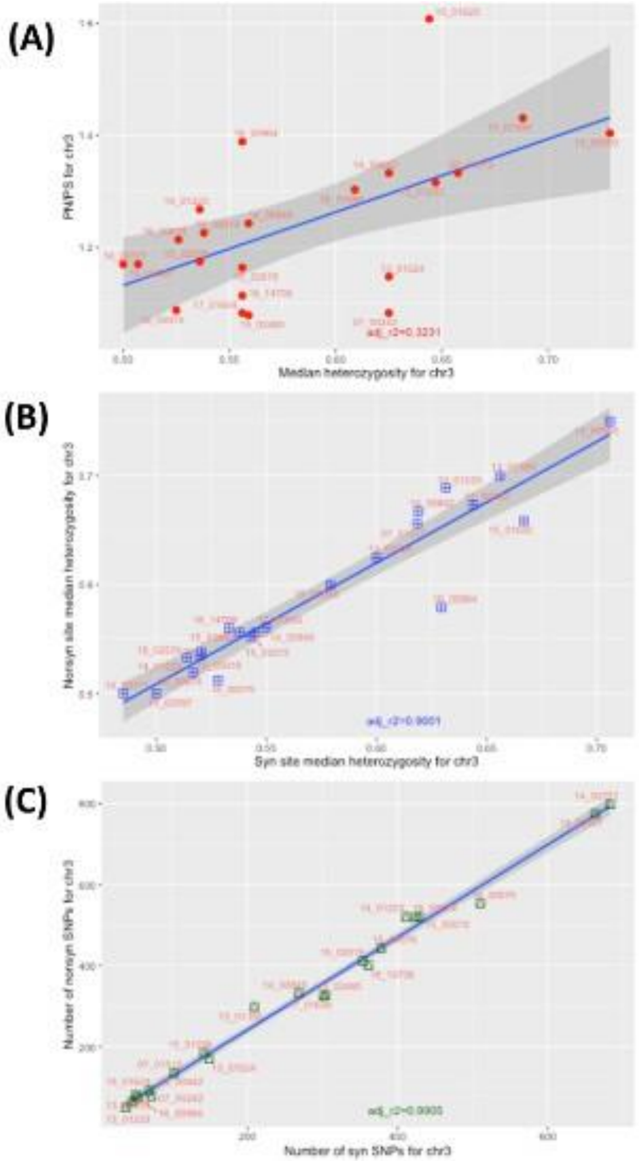

Figure 4. For chromosome 4 (like chromosome 22), 07_00242 had an exceptionally high PN/PS of 15.4 that was associated with complete homozygosity (A), but not a difference in nonsynonymous relative to synonymous site heterozygosity (B), whereas the high PN/PS was due to a high number of nonsynonymous SNPs (231 vs 15 for synonymous SNPs) (C). 14_00642 also had less heterozygosity and a higher relative number of nonsynonymous SNPs.

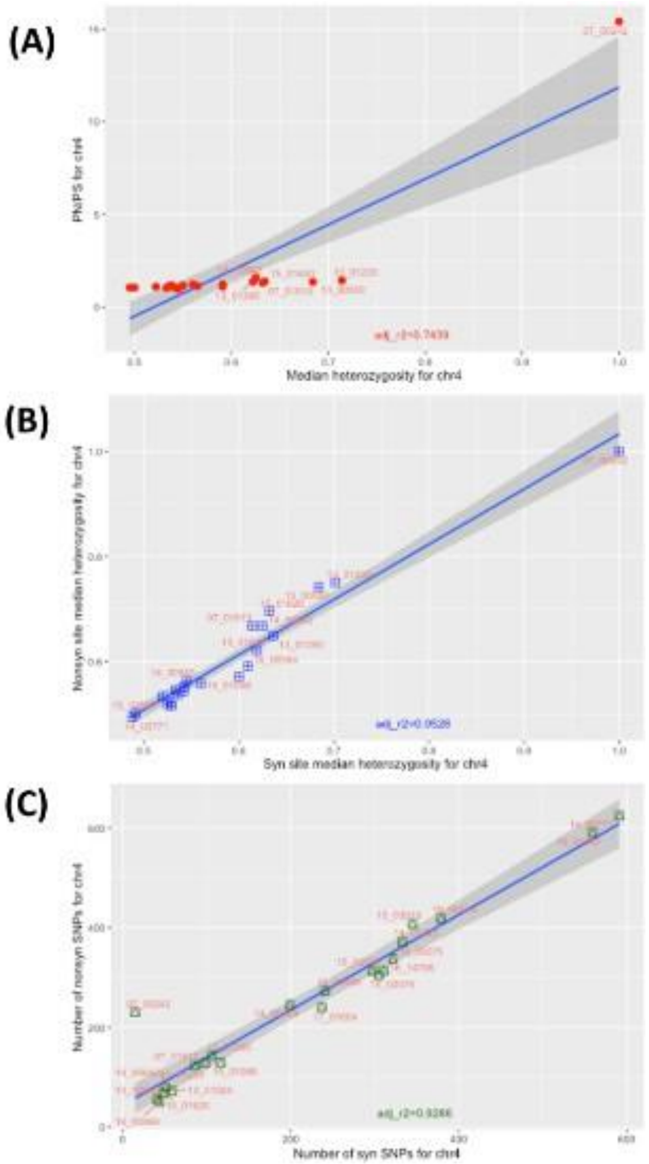

Figure 5. For chromosome 5, 14_00642's high PN/PS rate was associated with less heterozygosity (A), but not a higher nonsynonymous relative to synonymous site heterozygosity (B), but the high PN/PS was marginally due to a high number of nonsynonymous SNPs (C).

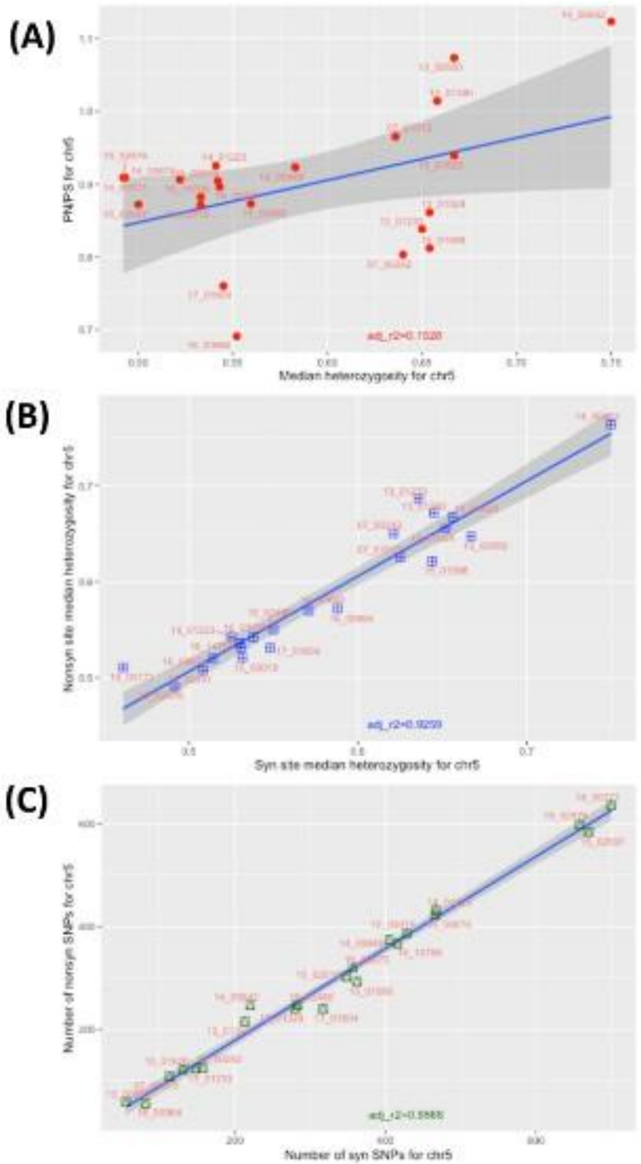

Figure 6. For chromosome 6, 16_00964's high PN/PS rate was not associated with less heterozygosity (A), not to a higher nonsynonymous relative to synonymous site heterozygosity (B), the high PN/PS was due to a high number of nonsynonymous SNPs (C).

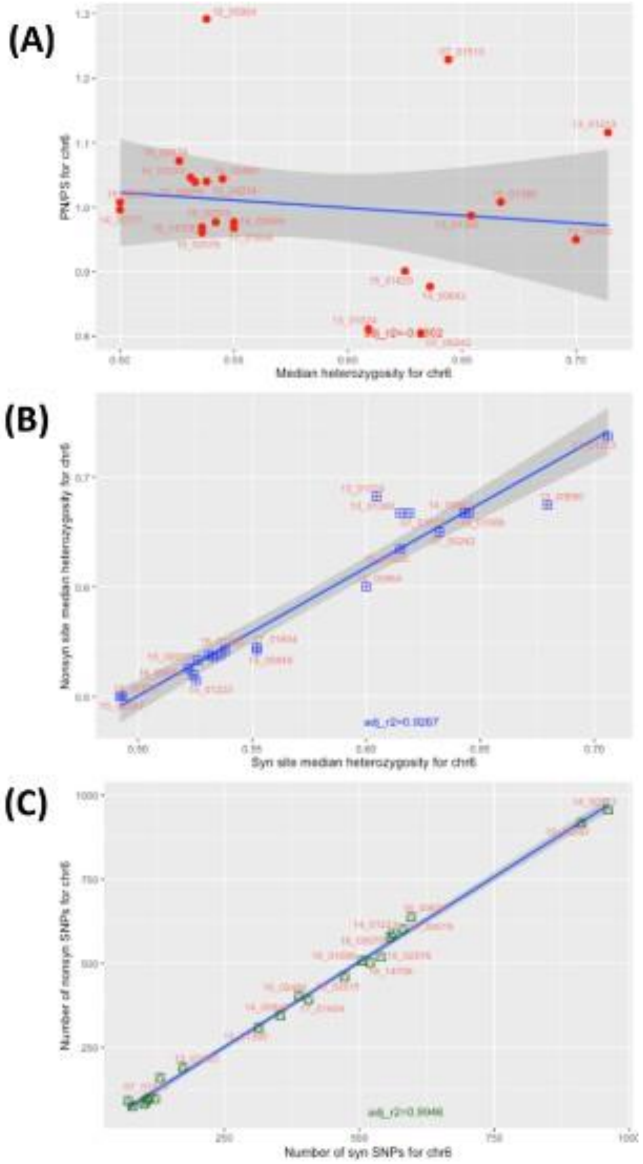

Figure 7. For chromosome 7, 15_01620's high PN/PS rate was associated with less heterozygosity (A), and a higher nonsynonymous relative to synonymous site heterozygosity (B), but not a higher number of nonsynonymous SNPs (C).

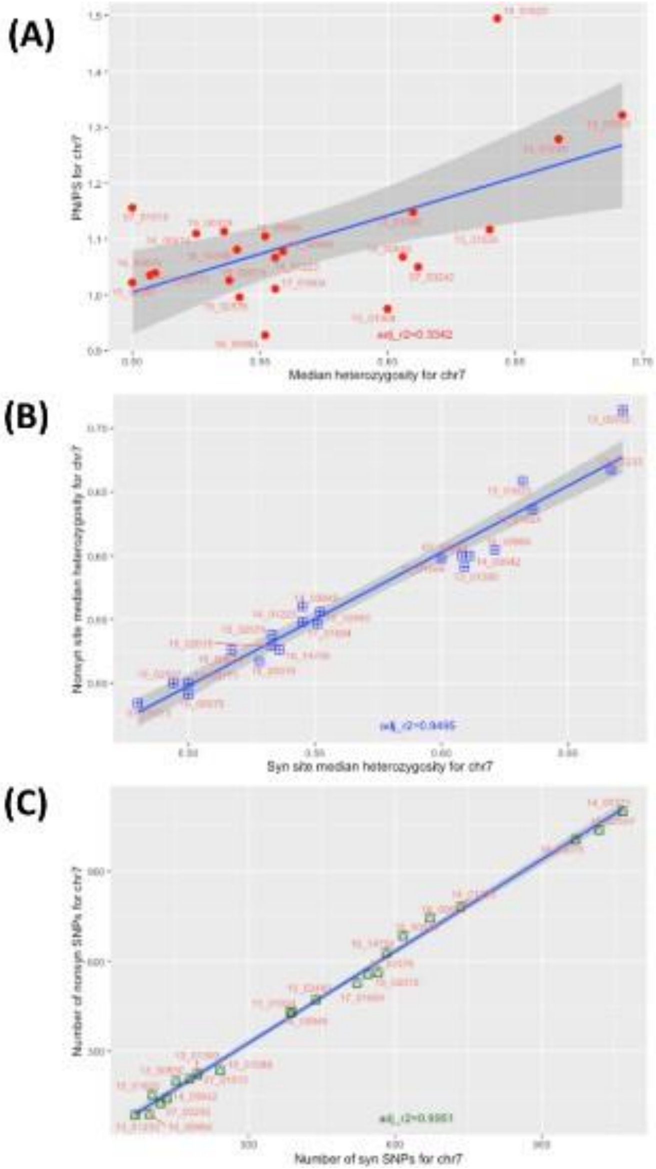

Figure 8. For chromosome 8, 13_00550's higher PN/PS rate was associated with less heterozygosity (A), and a difference in the nonsynonymous relative to synonymous site heterozygosity (B), and a slightly higher number of nonsynonymous SNPs (C).

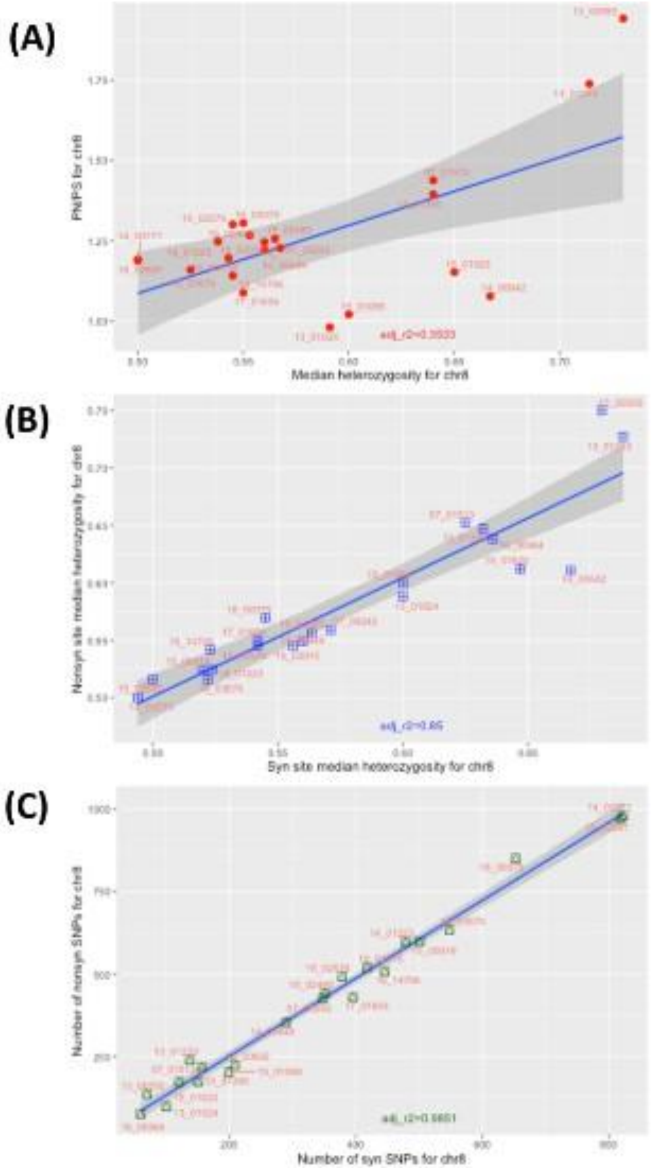

Figure 9. For chromosome 9, 13_00550's marginally higher PN/PS rate was associated with less heterozygosity (A), and a slight difference in the nonsynonymous relative to synonymous site heterozygosity (B), but not a higher number of nonsynonymous SNPs (C).

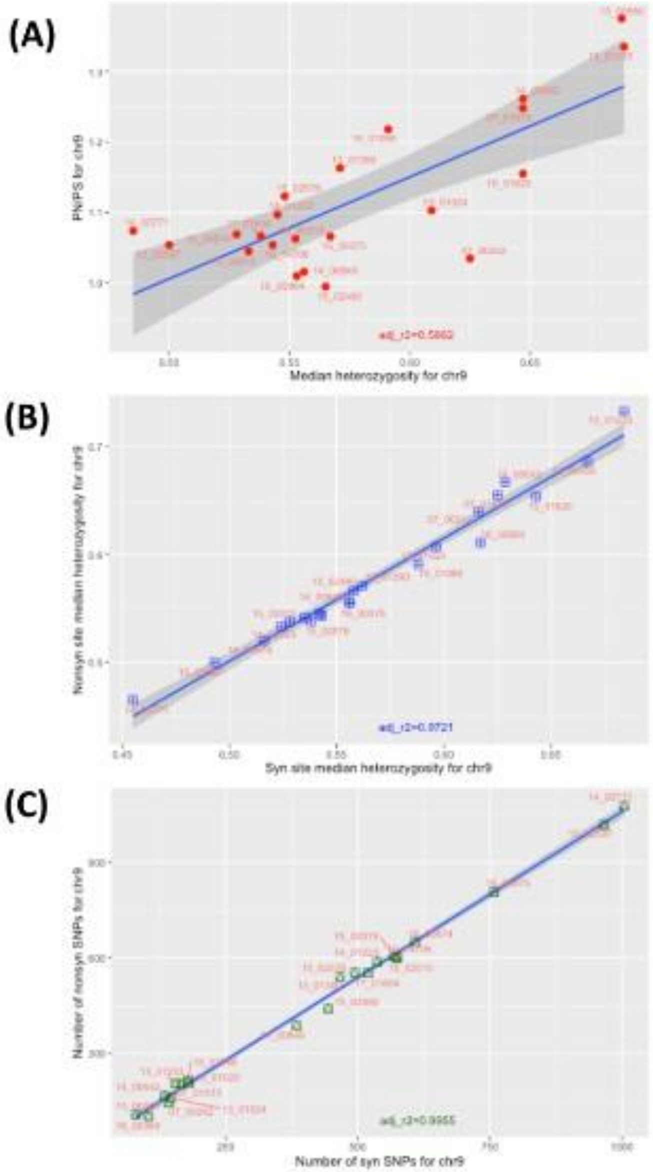

Figure 10. For chromosome 10, 14_00642 had a somewhat higher PN/PS rate and elevated median heterozygosity (A) that was not linked to elevated nonsynonymous relative to synonymous site heterozygosity (B), but perhaps due to a marginally higher number of nonsynonymous SNPs (C). 13_01233 14_00642 had a higher PN/PS rate (A) that was not linked to elevated nonsynonymous relative to synonymous site heterozygosity (B), not to a higher number of nonsynonymous SNPs (C).

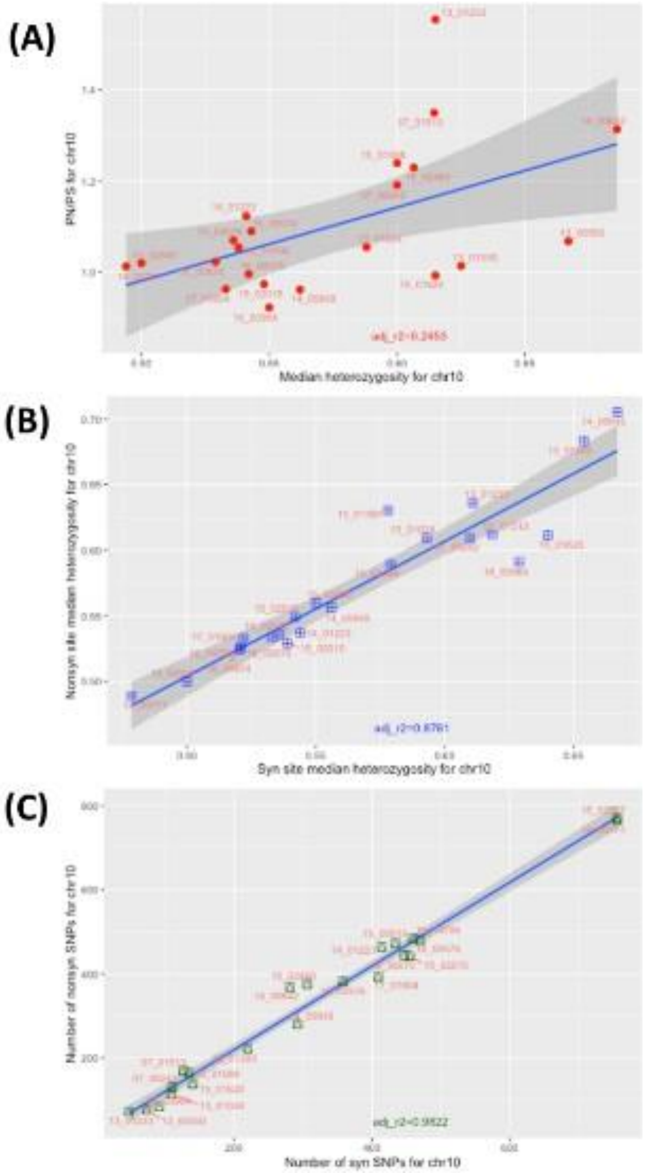

Figure 11. For chromosome 11, 07_01513's high PN/PS was associated with less heterozygosity (A), and a higher nonsynonymous relative to synonymous site heterozygosity (B), and also a high number of nonsynonymous SNPs (C).

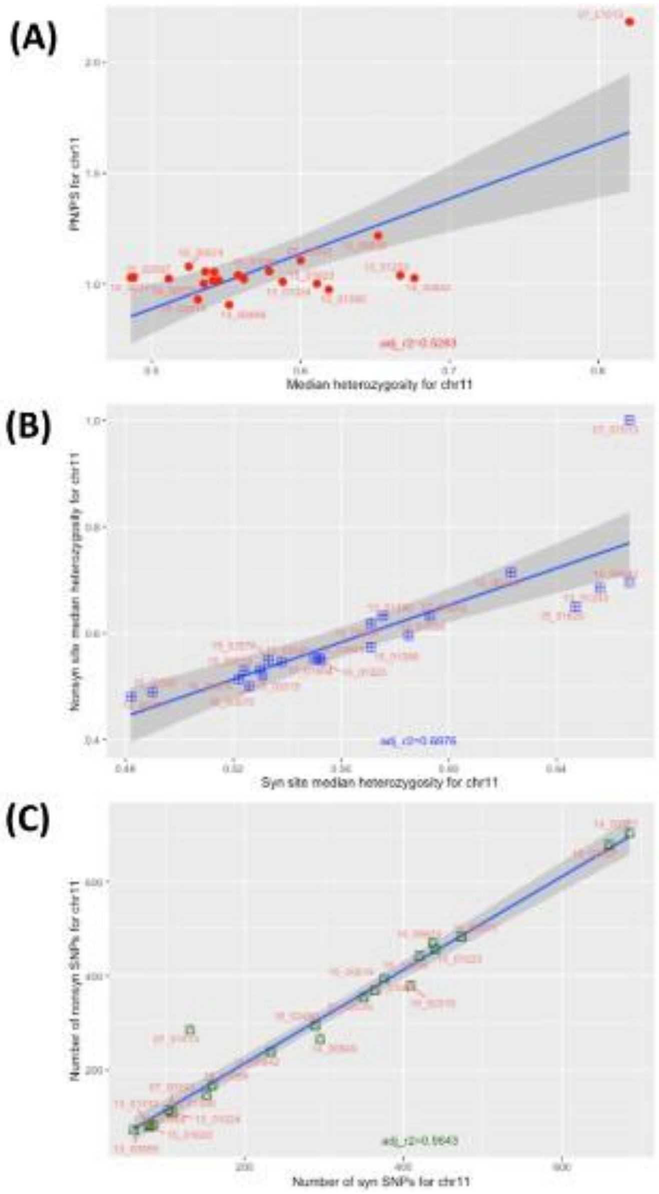

Figure 12. For chromosome 12, 15_02480's high PN/PS was associated with less heterozygosity (A), and a higher nonsynonymous relative to synonymous site heterozygosity (B), and also a high number of nonsynonymous SNPs (C).

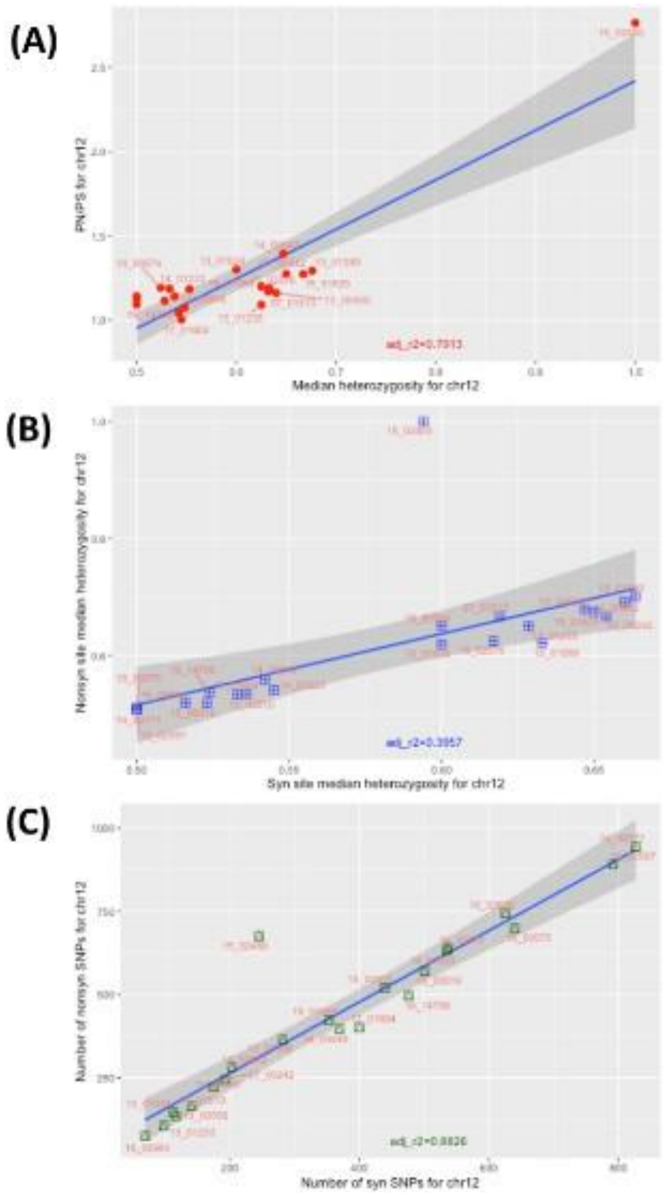

Figure 13. For chromosome 13, 07_00242 and 16_00964 had high PN/PS rates and markedly altered heterozygosity levels (A). This was linked to elevated nonsynonymous relative to synonymous site heterozygosity (B), and for 07_00242 only, a higher number of nonsynonymous SNPs (C). Both isolates came from patients originally from Iran.

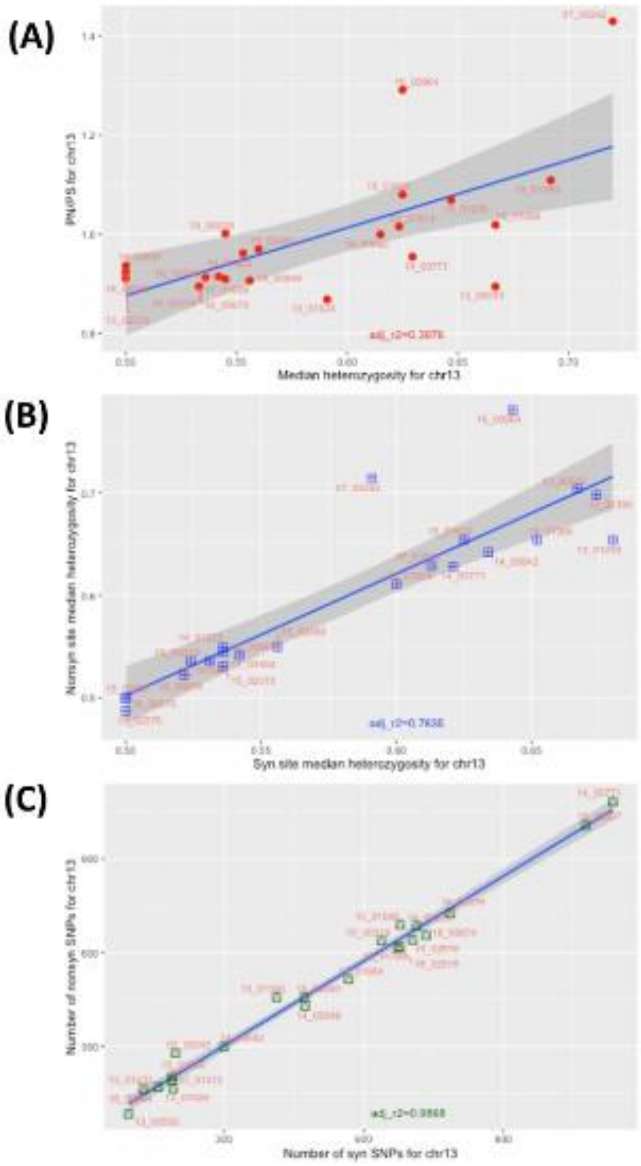

Figure 14. For chromosome 14, 13_00550's but not 14_00642's and 16_00964's was associated with less heterozygosity (A), and these had inconsistent differences in nonsynonymous relative to synonymous site heterozygosity (B), and for 14_00642 alone, the high PN/PS was due to a high number of nonsynonymous SNPs (C).

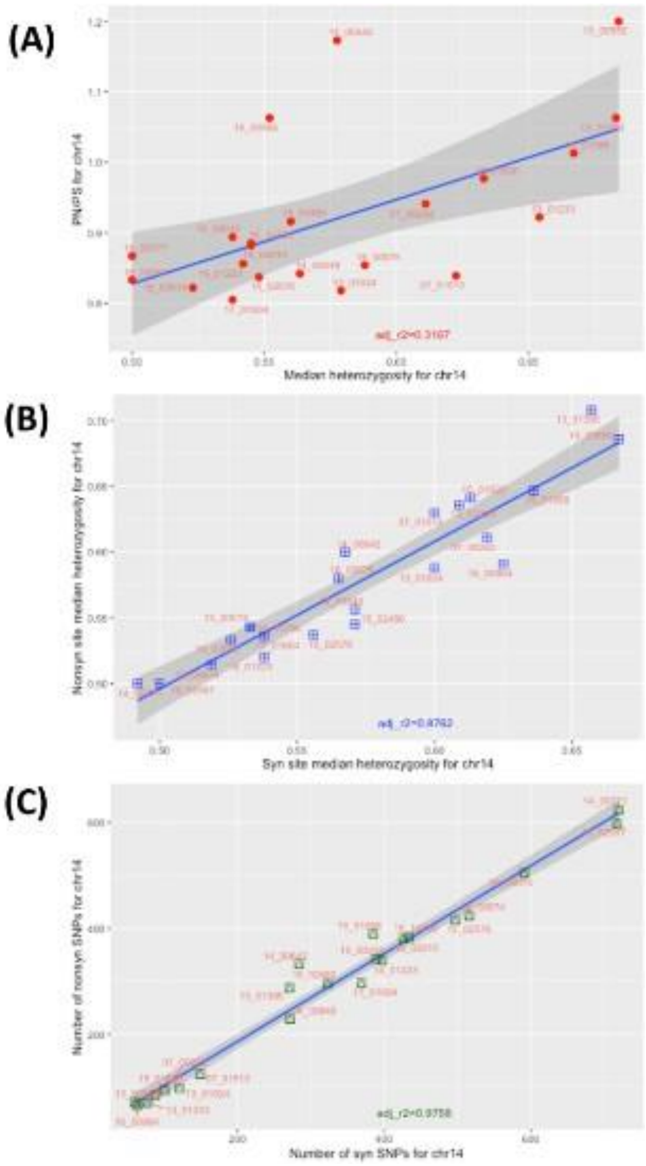

Figure 15. For chromosome 15, 07_01513's and 07_00242's higher PN/PS was associated with less heterozygosity (A), but not a higher nonsynonymous relative to synonymous site heterozygosity (B), nor a higher relative number of nonsynonymous SNPs (C).

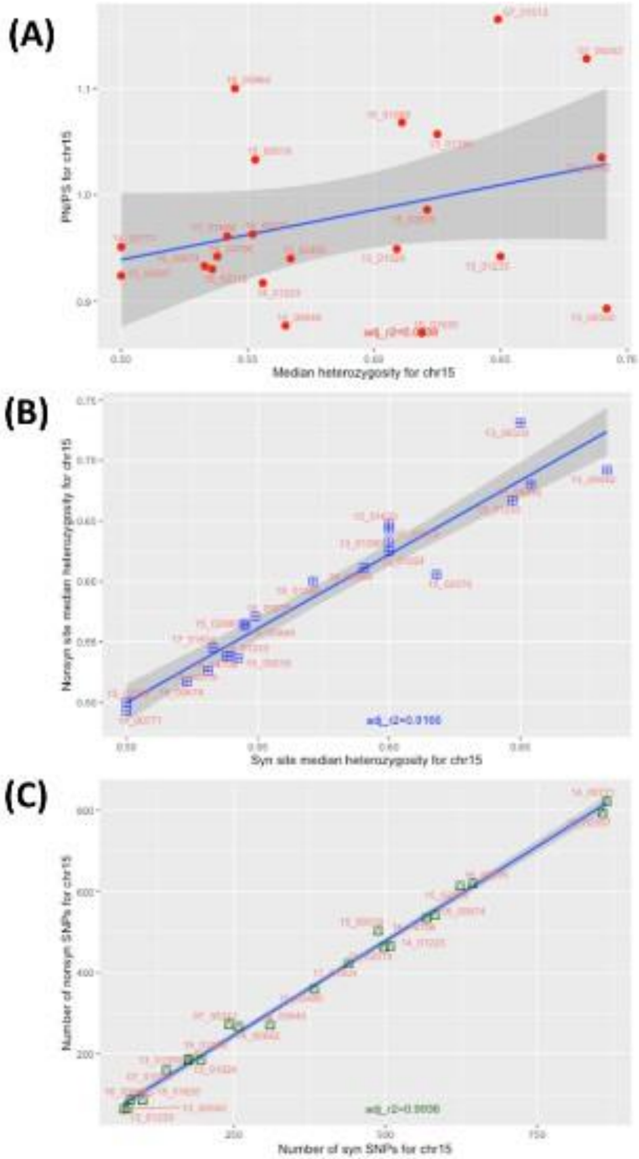

Figure 16. For chromosome 16, 07_01513's and 13_00550's but not 16_00964's higher PN/PS was associated with less heterozygosity (A), and for 07_01513 this was associated with a higher nonsynonymous relative to synonymous site heterozygosity (B), nor a higher relative number of nonsynonymous SNPs (C).

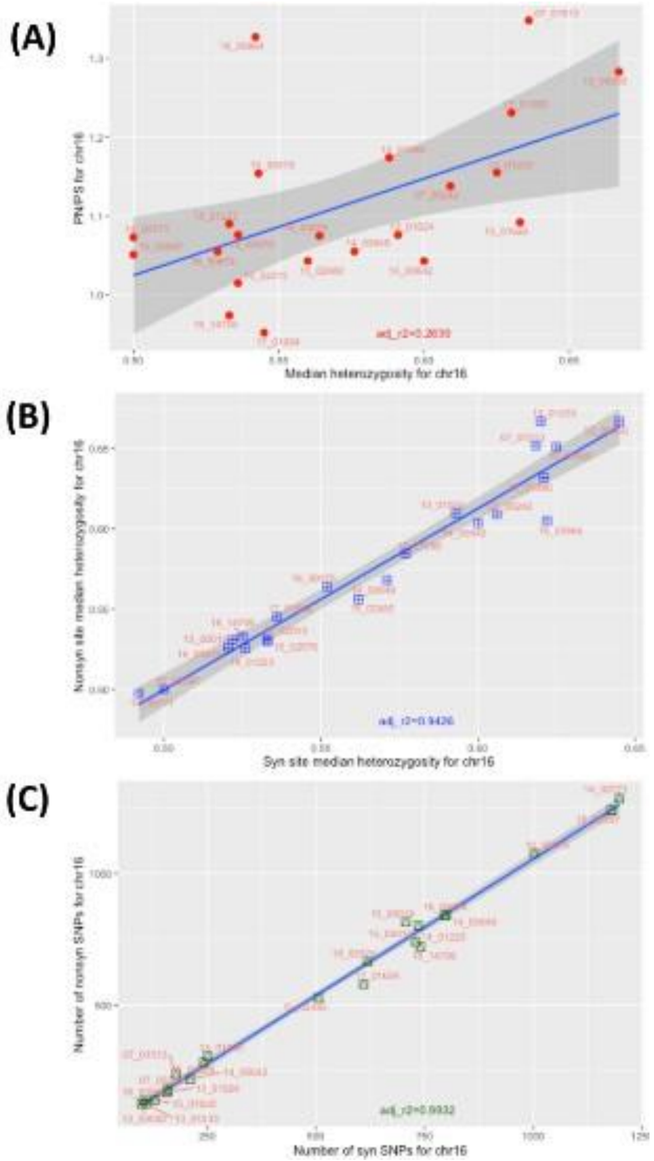

Figure 17. For chromosome 17, 07_00242's higher PN/PS rate was associated with marginally less heterozygosity (A), and a higher nonsynonymous relative to synonymous site heterozygosity (B), but not a higher relative number of nonsynonymous SNPs (C).

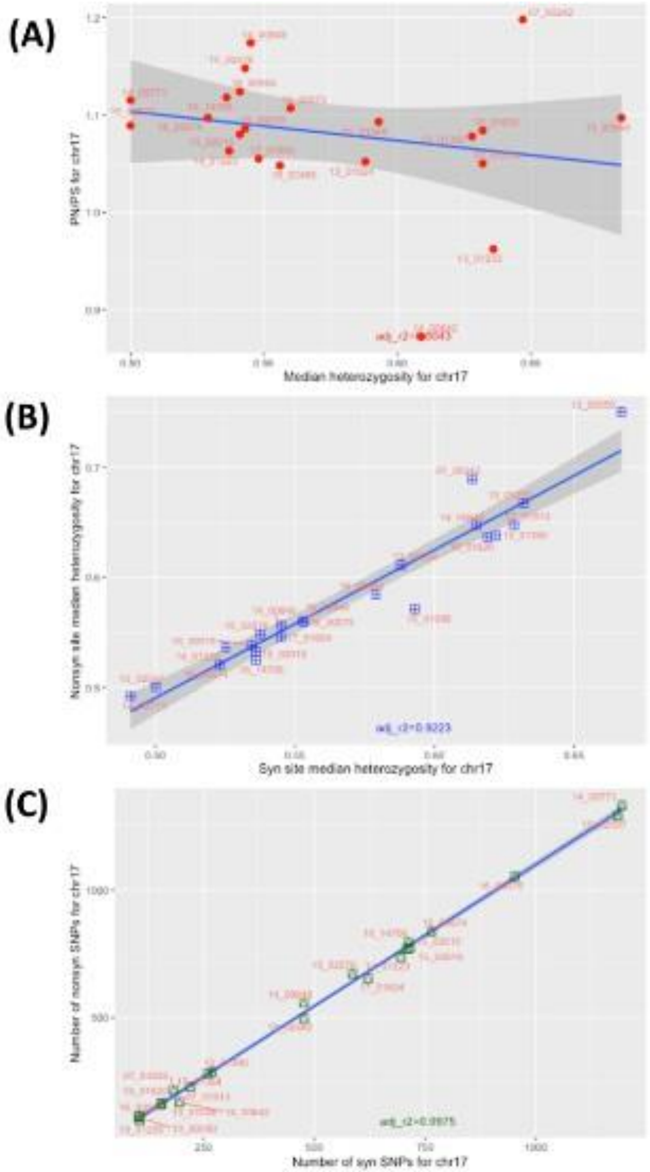

Figure 18. For chromosome 18, 07_01513's and 07_00242's higher PN/PS was associated with less heterozygosity (A), and to an extent with a higher nonsynonymous relative to synonymous site heterozygosity (B), but not a higher relative number of nonsynonymous SNPs (C).

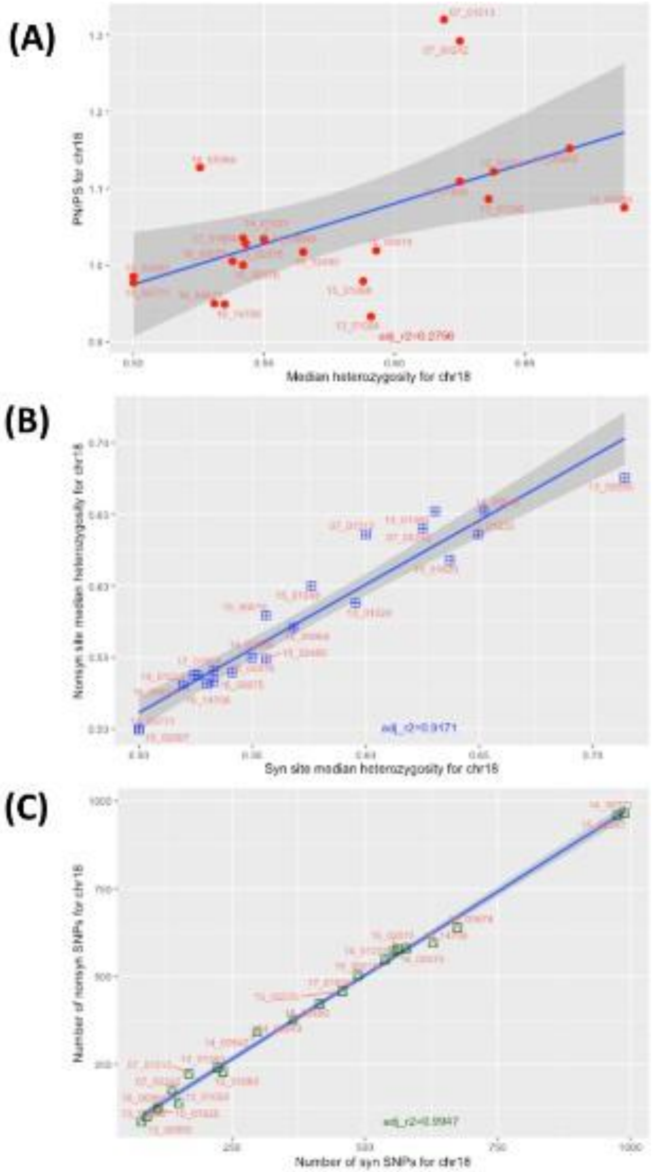

Figure 19. For chromosome 19, 13_01390's but not 15_02576's high PN/PS rate was associated with less heterozygosity (A), andfor both there were no differences in nonsynonymous relative to synonymous site heterozygosity (B), nor a higher relative number of nonsynonymous SNPs (C).

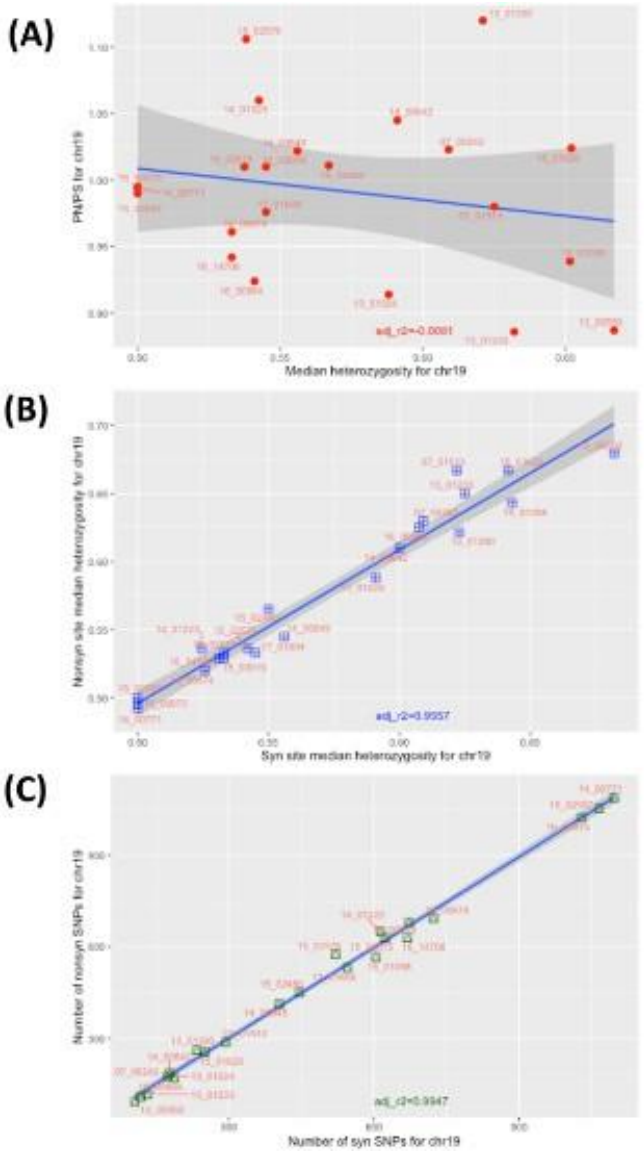

Figure 20. For chromosome 20, 13_00550's high PN/PS rate was associated with less heterozygosity (A), and elevated nonsynonymous relative to synonymous site heterozygosity (B), but this high PN/PS was not due to a high number of nonsynonymous SNPs (C).

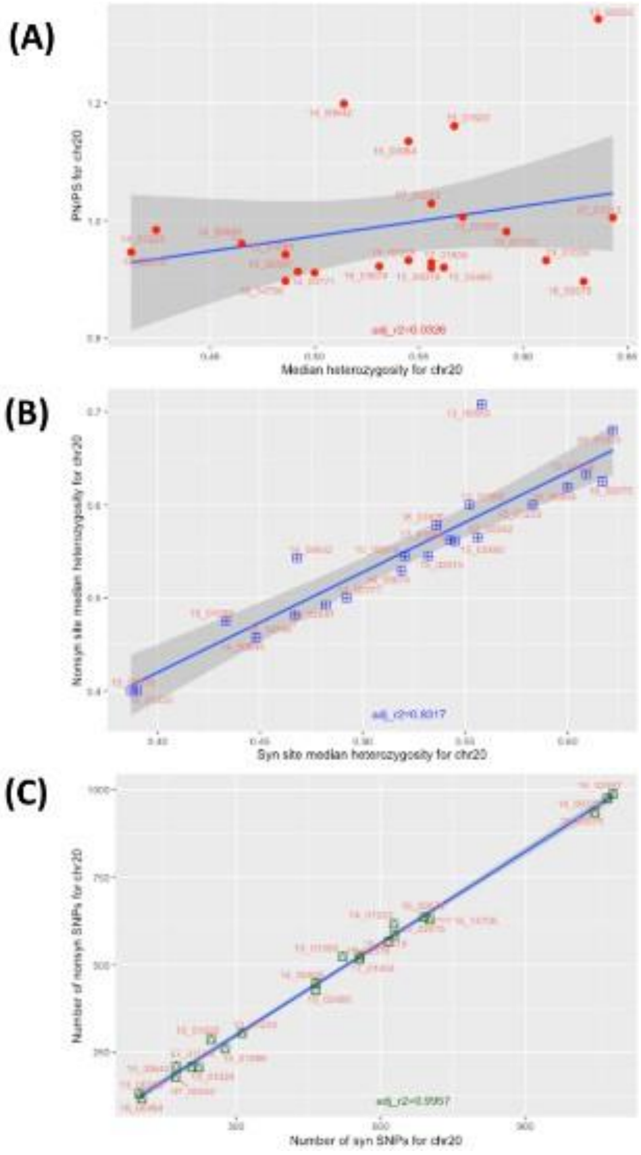

Figure 21. For chromosome 21, 13_00550's high PN/PS rate was associated with differing heterozygosity levels (A) and for 13_00550 this was associated with a somewhat higher nonsynonymous relative to synonymous site heterozygosity (B), but a higher number of nonsynonymous SNPs (C).

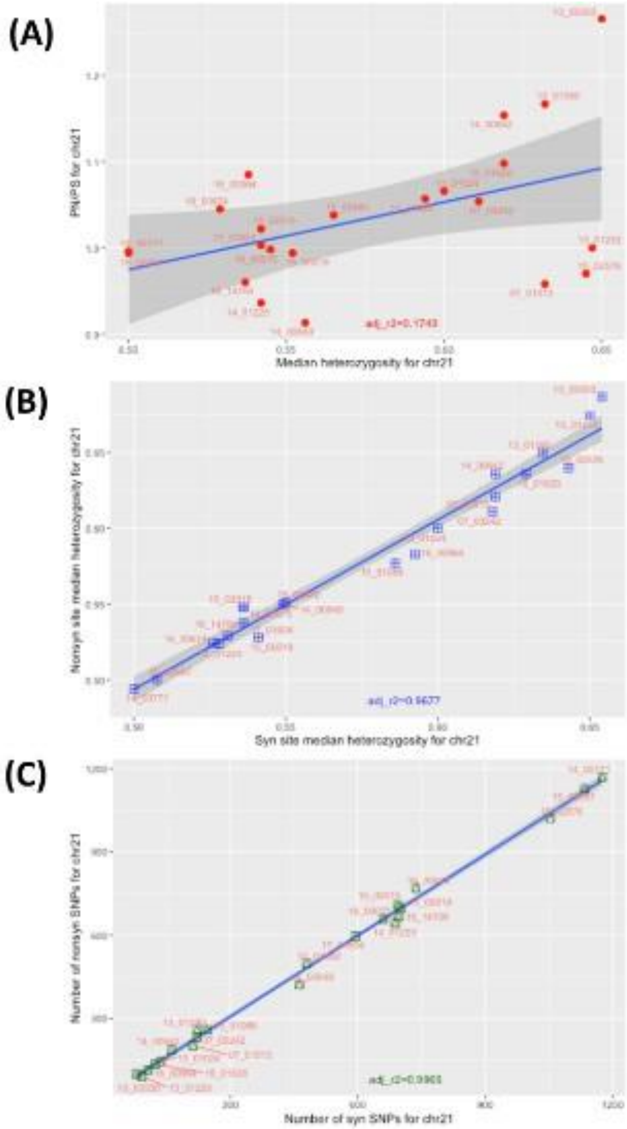

Figure 22. For chromosome 22 (like chromosome 4), 07_00242 had an exceptionally high PN/PS of 18.4 that was associated with complete homozygosity (A), but not a difference in nonsynonymous relative to synonymous site heterozygosity (202 vs 11 for synonymous sites) (B), whereas the high PN/PS was due to a high number of nonsynonymous SNPs (C). 14_00642 also had less heterozygosity and a higher relative number of nonsynonymous SNPs.

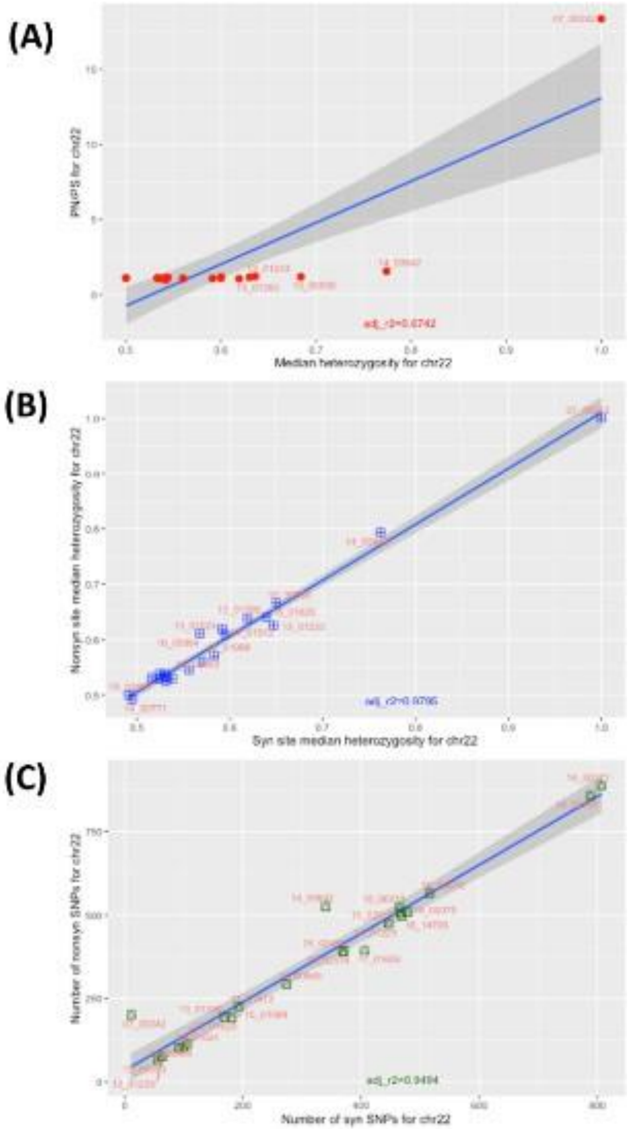

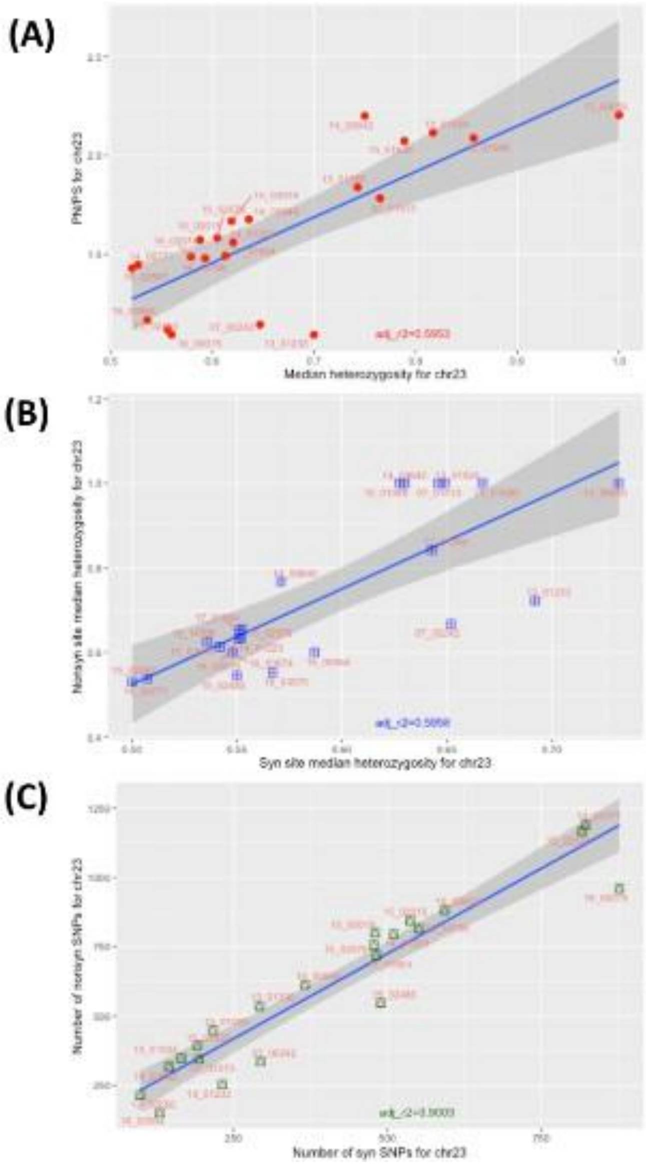

Figure 23. For chromosome 23, 07_00242, 13_01233, 15_02480, 16_00075 and 16_00964 had substantially lower PN/PS rates across a range of heterozygosity levels (A). This was linked to reduced nonsynonymous relative to synonymous site heterozygosity (B), and a dearth of nonsynonymous SNPs (C). All five isolates came from patients originally from Iran or Afghanistan, and previously had a substantial long run of homozygosity (LROH) at <250 Kb on this chromosome. The average PN/PS rate across all 22 samples for this chromosome was higher than the other chromosome's (1.50 vs 1.06+-0.14, 99.9th percentile).

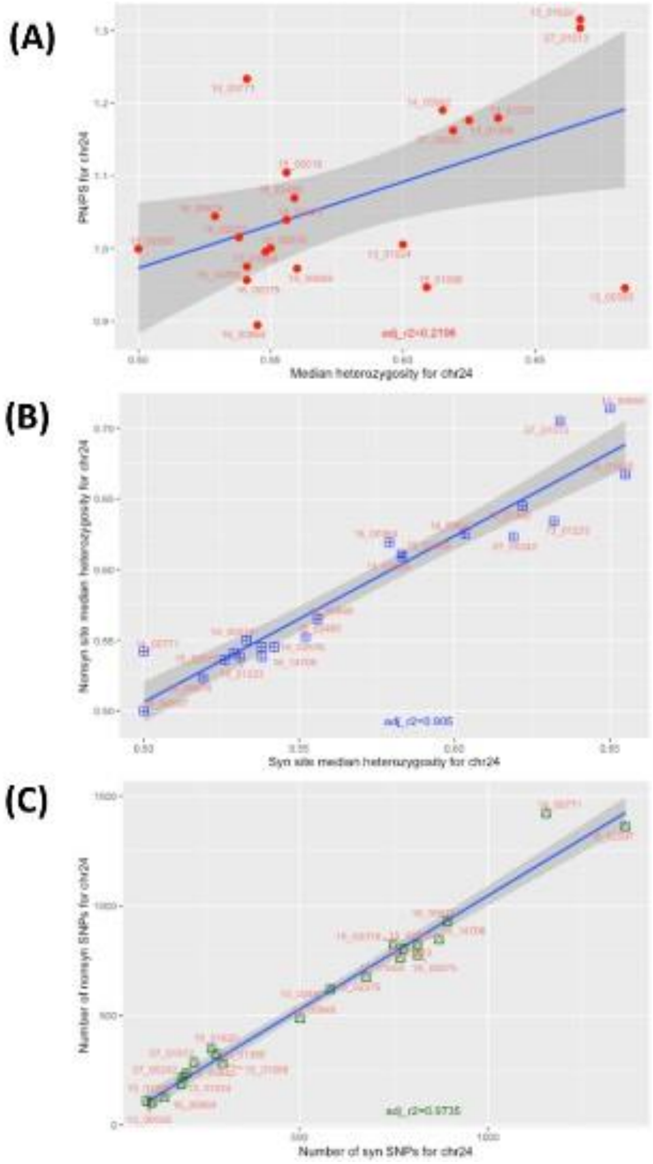

Figure 24. For chromosome 24, 14_00771's high PN/PS was not associated with less heterozygosity whereas it was for 07_01513 and 15_01620 (A), and this was associated with a higher nonsynonymous relative to synonymous site heterozygosity in 07_01513 only (B), and a higher number of nonsynonymous SNPs in 14_00771 only (C).

Figure 25. For chromosome 25, 13_00550's but not 14_00849's high PN/PS rate was associated with differing heterozygosity levels (A) and for 13_00550 this was associated with higher nonsynonymous relative to synonymous site heterozygosity (B). For both, this was not due to a high number of nonsynonymous SNPs (C).

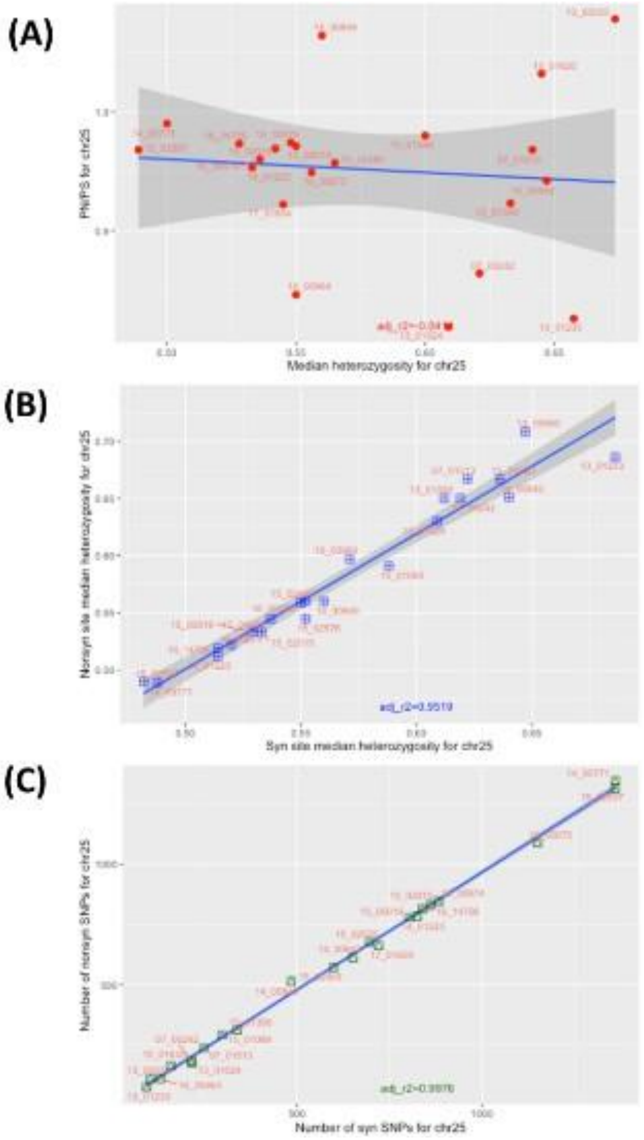

Figure 26. For chromosome 26, 14_00642's high PN/PS was associated with less heterozygosity (A), but not a change in nonsynonymous relative to synonymous site heterozygosity (B), nor was it due to a high number of nonsynonymous SNPs (C).

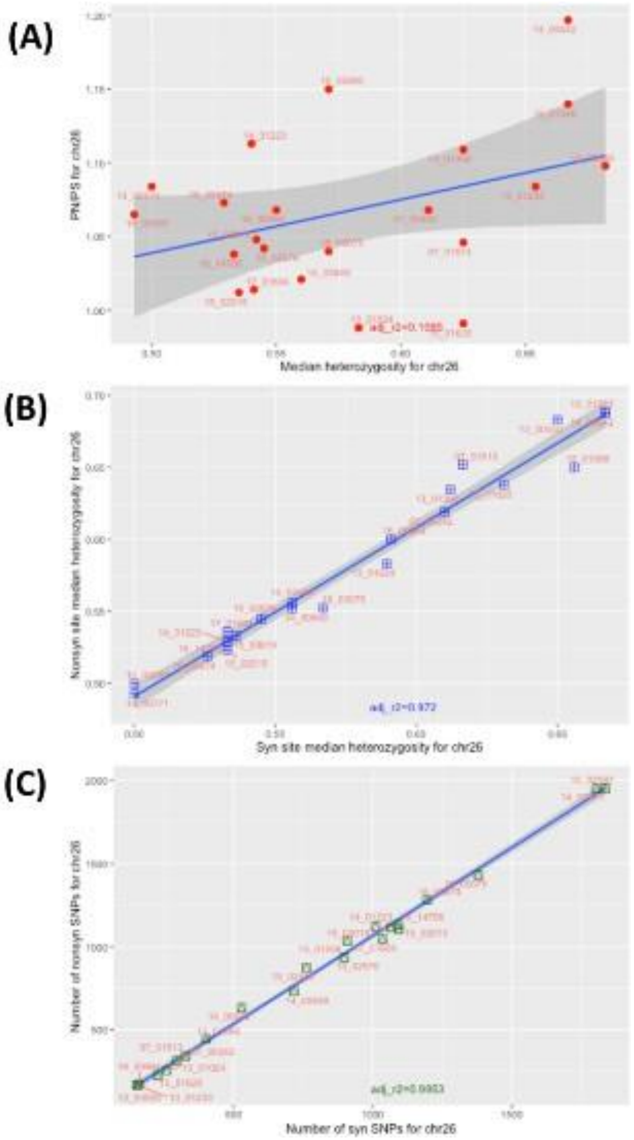

Figure 27. For chromosome 27, 13_01390's high PN/PS of 3.21 rate was associated with less heterozygosity (A), and although there were no differences in nonsynonymous relative to synonymous site heterozygosity (575 vs 179 for synonymous sites) (B), the high PN/PS was due to a high number of nonsynonymous SNPs (C).

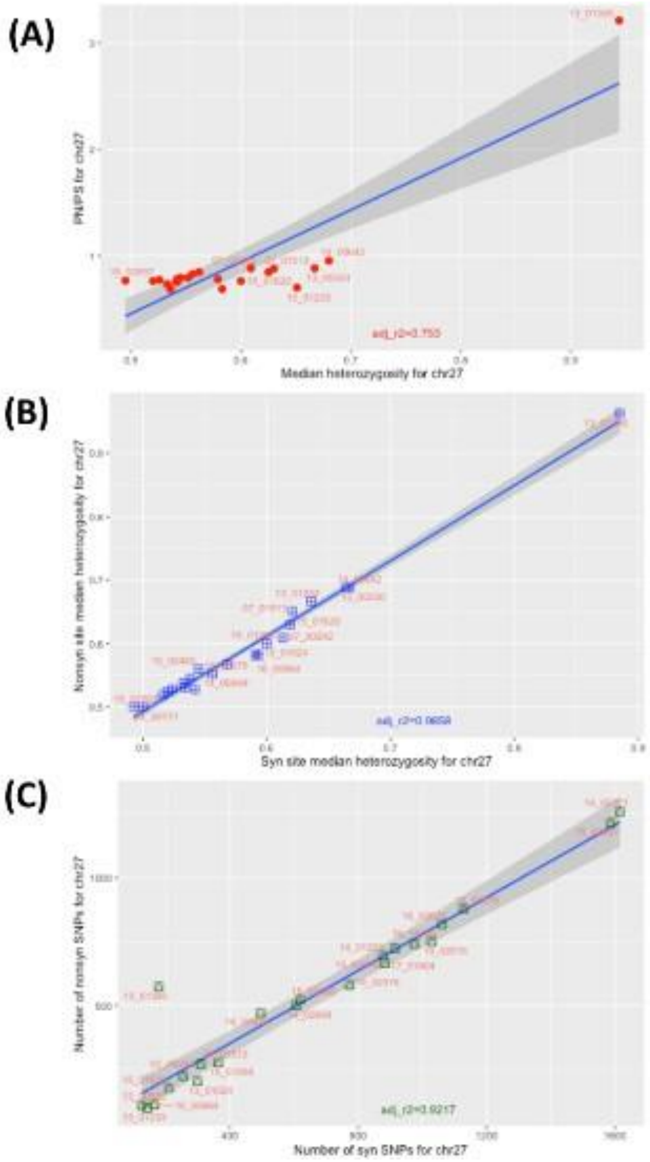

Figure 28. For chromosome 28, 16_00964's high PN/PS of 3.86 was associated with less heterozygosity (A), and although there were no differences in nonsynonymous relative to synonymous site heterozygosity (B), the high PN/PS was due to a high number of nonsynonymous SNPs (703 vs 182 for synonymous sites) (C). The latter higher nonsynonymous SNP rate was also present for 15_02480 and 14_00771 as well (C).

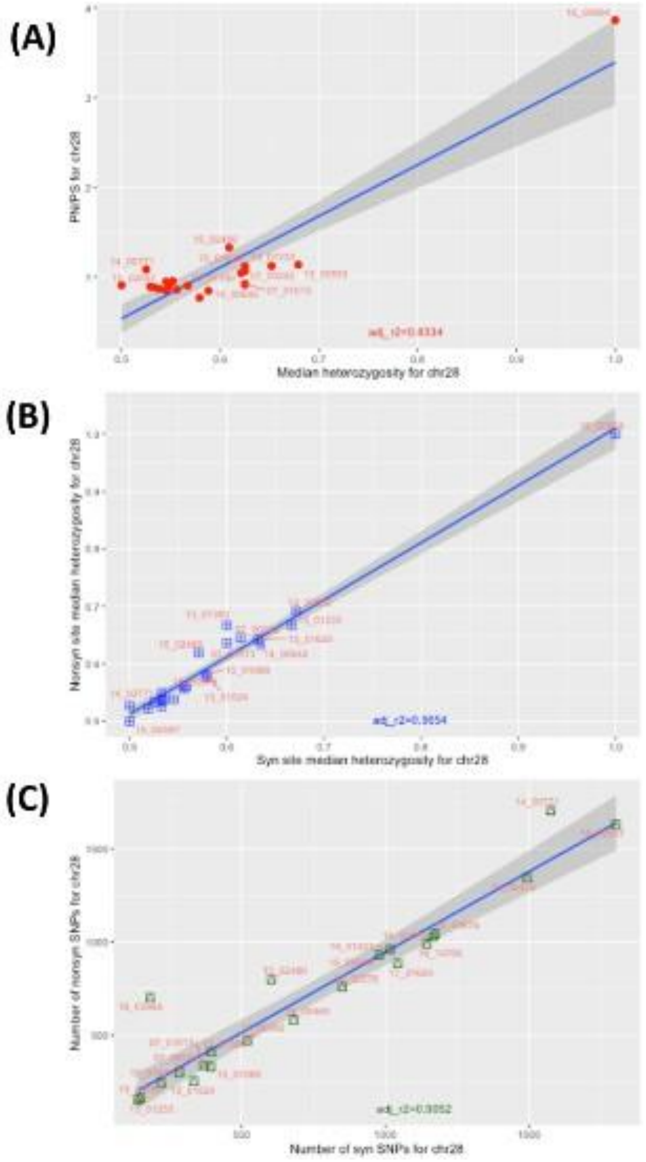

Figure 29. For chromosome 29, 07_00242 and 16_00964 had high PN/PS rates (4.99 and 3.12) and markedly reduced heterozygosity levels (A). This was linked to elevated nonsynonymous relative to synonymous site heterozygosity (B), and a higher number of nonsynonymous SNPs (569 vs 114 for synonymous sites for 07_00242, and 483 vs 16_00964 for synonymous sites for) (C). Both isolates came from patients originally from Iran and previously had a substantial long run of homozygosity (LROH) at >490 Kb on this chromosome.

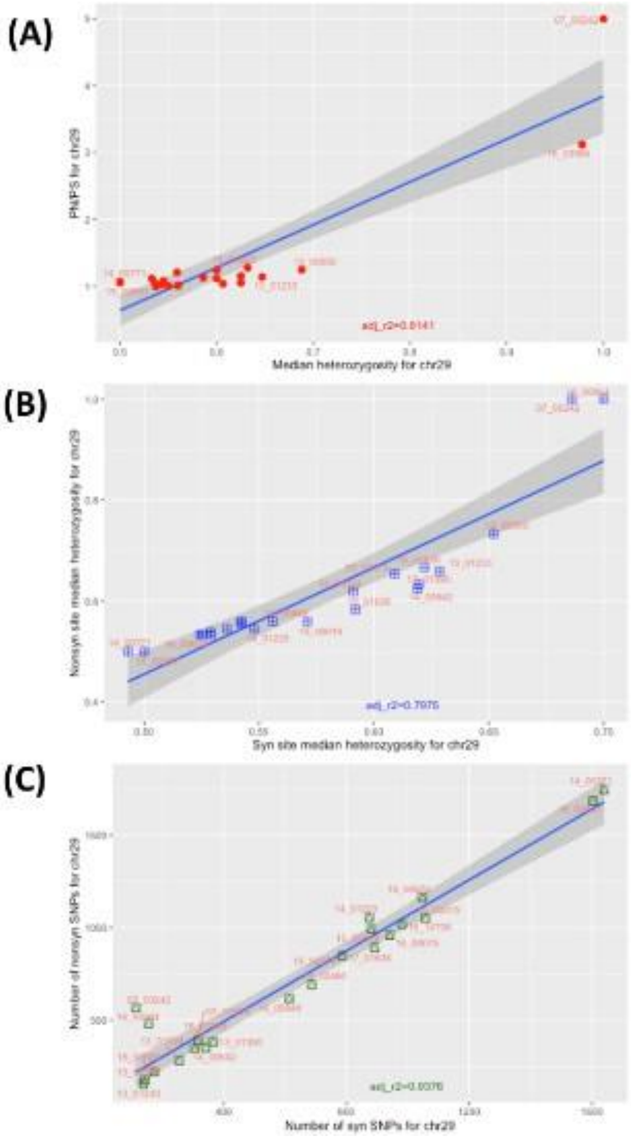

Figure 30. For chromosome 30, there were no associations of PN/PS rates with heterozygosity (A), and no differences in the nonsynonymous relative to synonymous site heterozygosity (B), nor in the relative number of nonsynonymous SNPs (C).

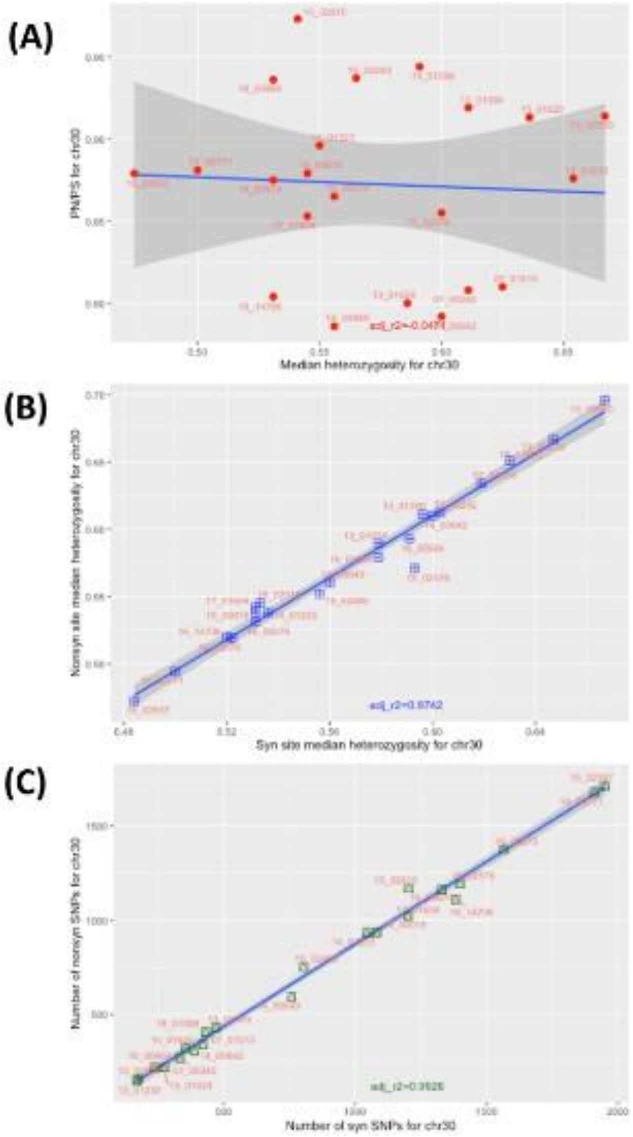

Figure 31. For chromosome 31, 15_01620, 16_00964, 13_00550 and 13_01233 had high PN/PS rates but differing median heterozygosity levels (A). This was not linked to elevated nonsynonymous relative to synonymous site heterozygosity (B), nor a high number of nonsynonymous SNPs (C).

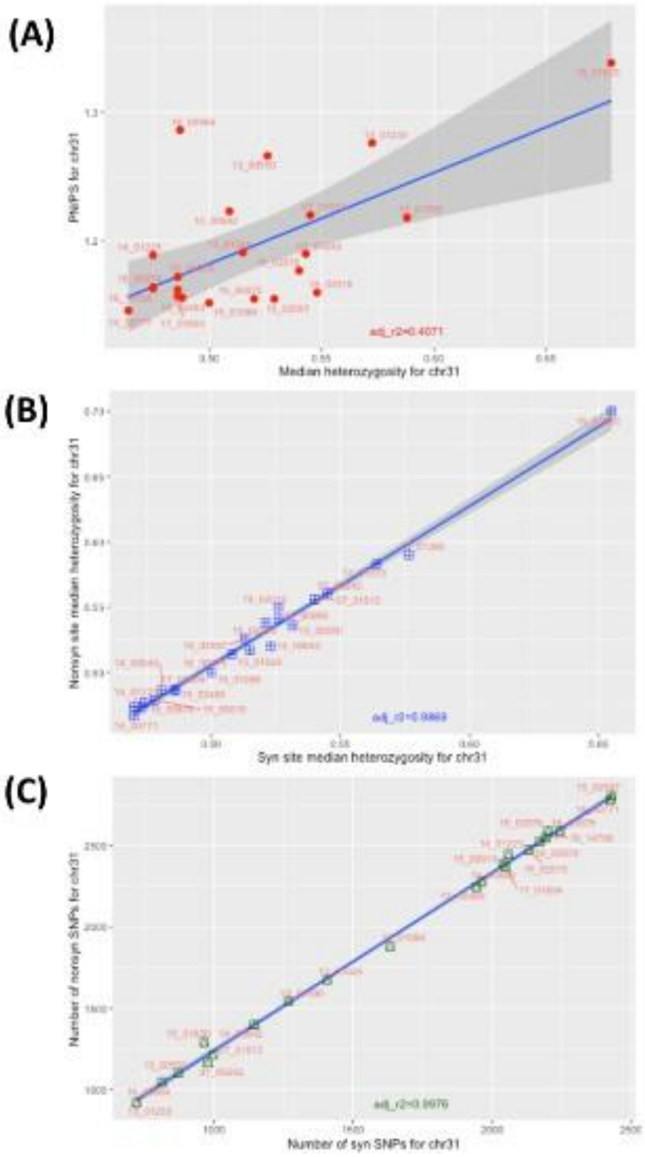

Figure 32. For chromosome 32, 07_00242's high PN/PS rate (14.79) was associated with less heterozygosity whereas and 15_02015's elevated homozygosity was not (A). For the latter, this was associated with a higher nonsynonymous relative to synonymous site heterozygosity, but not for 07_00242 (B). For both, the high PN/PS was due to a high number of nonsynonymous SNPs (1139 vs 77 for synonymous sites for 07_00242) (C).

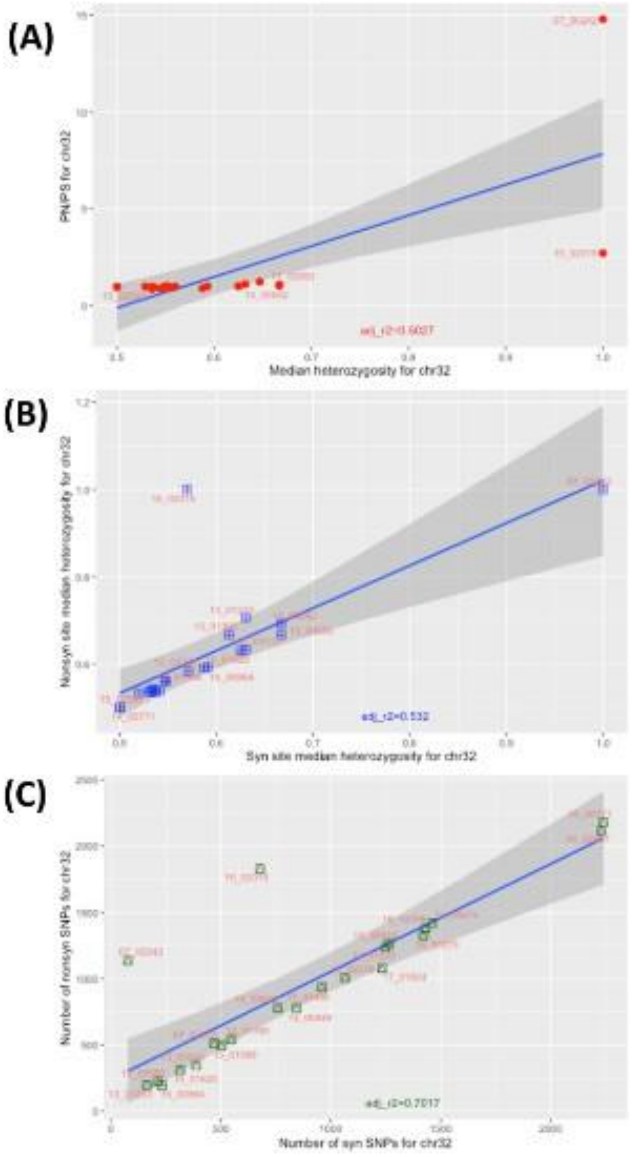

Figure 33. For chromosome 33, 07_00242's high PN/PS rate was associated with less heterozygosity (A), which was associated with a higher nonsynonymous relative to synonymous site heterozygosity (B), and the high PN/PS was due to a high number of nonsynonymous SNPs (C).

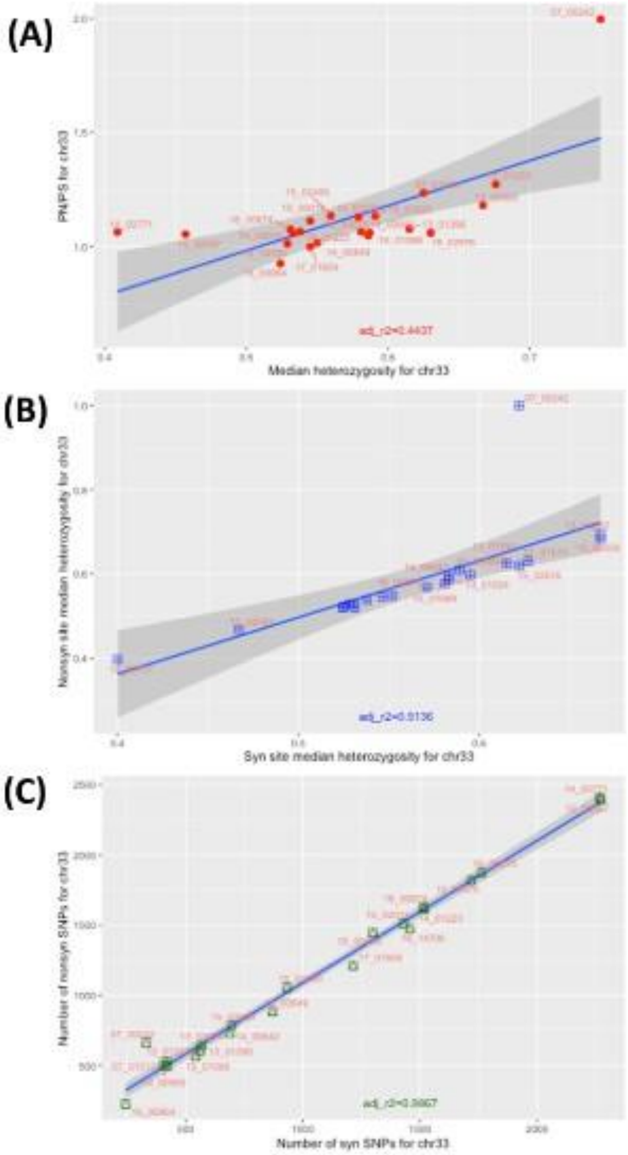

Figure 34. For chromosome 34, 13_00550's high PN/PS rate was associated with less heterozygosity (A), but not an elevated nonsynonymous relative to synonymous site heterozygosity (B), and not a higher number of nonsynonymous SNPs (C).

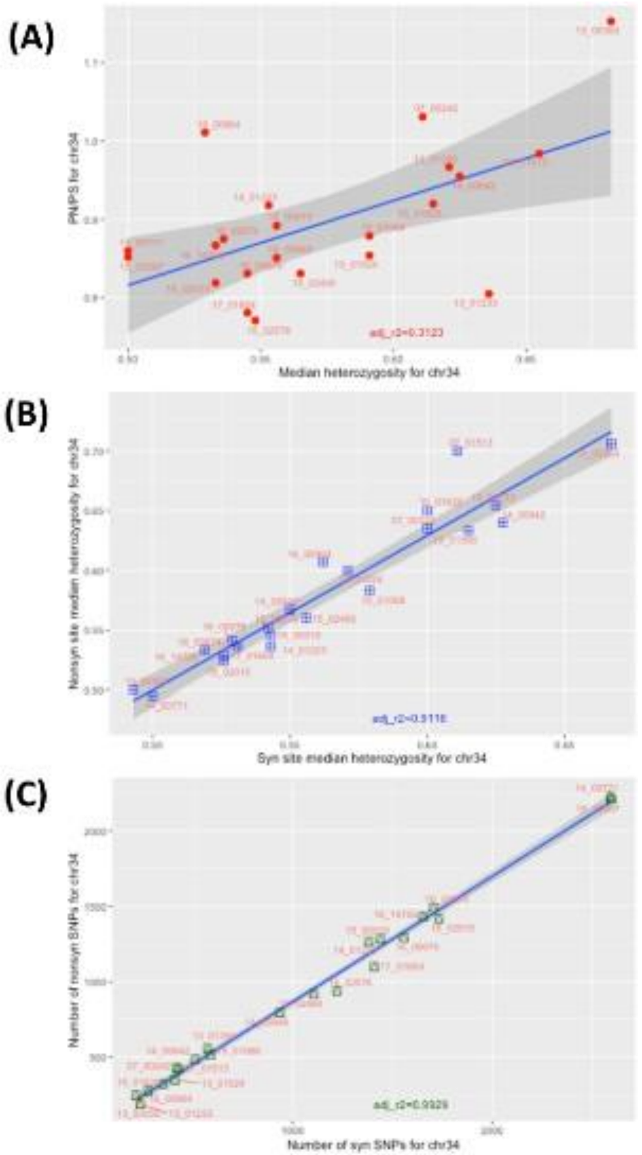

Figure 35. For chromosome 35, 13_00550's high PN/PS rate was associated with less heterozygosity (A), but not an elevated nonsynonymous relative to synonymous site heterozygosity (B), and was possibly due to a slightly higher number of nonsynonymous SNPs (C).

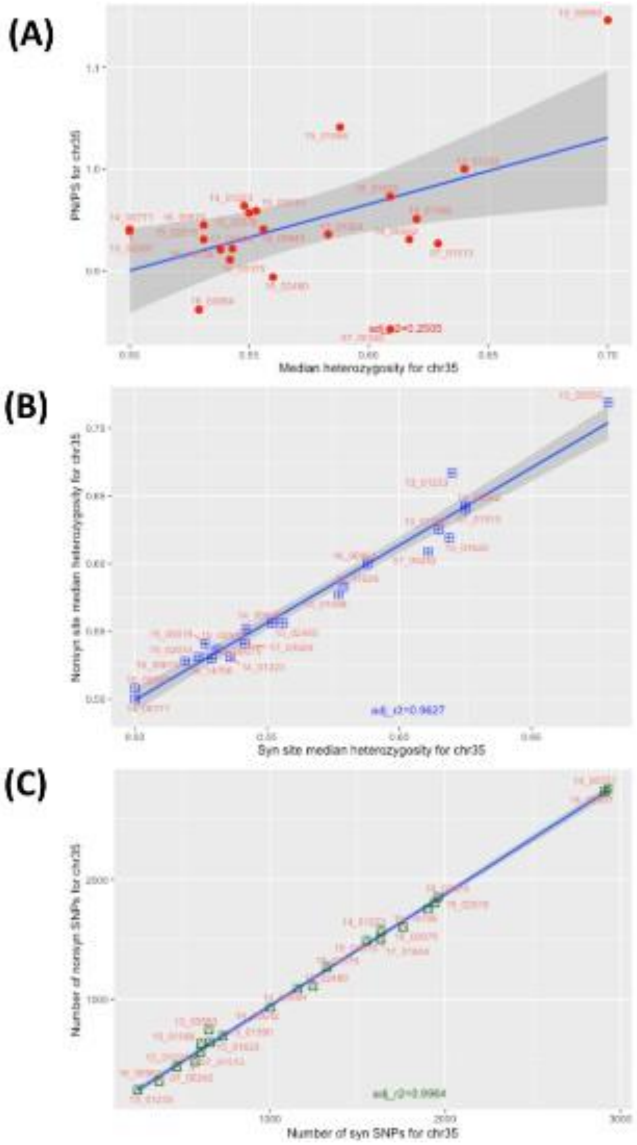

Figure 36. For chromosome 36, 07_00242 and 16_00964 had high PN/PS rates but differing median heterozygosity levels (A). For both, this was linked to elevated nonsynonymous relative to synonymous site heterozygosity (B), and not a high number of nonsynonymous SNPs (C).

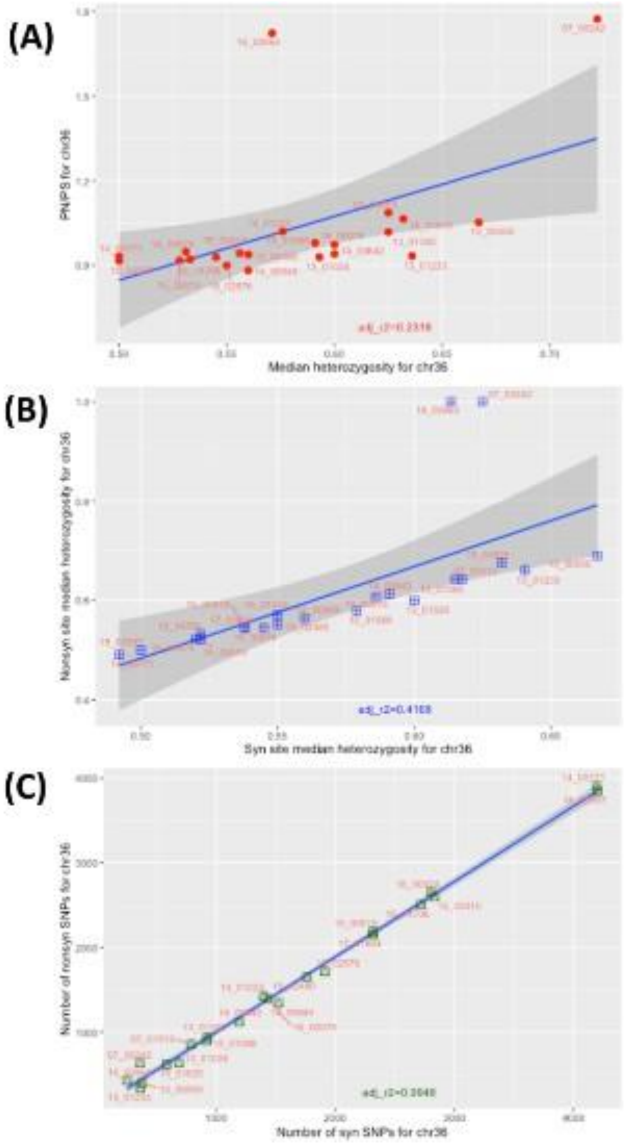

Supplement: S3 Fig — For each chromosome, the association between (A) PN/PS rates (y-axis) with heterozygosity (x-axis) (each sample is a red dot); (B) the synonymous site heterozygosity (x-axis) versus the nonsynonymous site heterozygosity (y-axis) (each sample is a blue square); and (C) the numbers of synonymous SNPs (x-axis) versus the numbers of nonsynonymous SNPs (y-axis). The blue line per plot is a linear model of the best fit between the two variables across the samples, with the 95% confidence interval shown by the grey area. The adjusted r2 value for this model is shown on each plot. (PDF) [file pone.0310821.s003.pdf]
