# Supplementary material for: Genetic coping mechanisms observed in Leishmania tropica, from the Middle East region, enhance the survival of the parasite after drug exposure
Source: PLoS One. 2024 Dec 3;19(12):e0310821. doi: 10.1371/journal.pone.0310821 (PMC11614225; doi:10.1371/journal.pone.0310821)
Supplement: S1 Table — (DOCX) [file pone.0310821.s008.docx]

**S1 Table**

|  | Gene id | Gene name | Function prediction | Previous findings |
| --- | --- | --- | --- | --- |
| H  locus | LmjF.23.2.000240 | Terbinafine resistance gene (HTBF), (YIP1) | Yip1 domain containing protein | Antimonial (R) The H locus is associated with metal resistance and is frequently amplified in drug resistant *Leishmania* strains (genes YIP1, MRPA, PTR1). (a, b) |
|  | LmjF.23.2.000210*, LmjF.23.2.000220*, LmjF.23.2.000250* | P-glycoprotein A (MRPA);  pentamidine resistance protein 1 | ATP-binding cassette (ABC) transporter, ABC- thiol transporter | Antimonial (R). An increased expression of MRPA, due to gene amplification, are seen in antimony resistant *Leishmania* strains. (a, b, c) |
|  | LmjF.23.2.000260 |  | argininosuccinate synthase  - putative | Antimonial (R) (c, d) |
|  | LmjF.23.2.000270 | Pteridine reductase 1 (PTR1) | short chain dehydrogenase/Enoyl- (Acyl carrier protein) reductase | Antimonial (R). see above on the H locus (a, b) |

* homologs, a. Callhan & Beverly 1991 b. Dias et al 2007 c. Leprohon et al 2009 d. Grondin et al 1993
