# Supplementary material for: Genetic coping mechanisms observed in Leishmania tropica, from the Middle East region, enhance the survival of the parasite after drug exposure
Source: PLoS One. 2024 Dec 3;19(12):e0310821. doi: 10.1371/journal.pone.0310821 (PMC11614225; doi:10.1371/journal.pone.0310821)
Supplement: S3 Table — (DOCX) [file pone.0310821.s010.docx]

**S3 Table**

| **Gene id** | **Function prediction** | **Previous findings** |
| --- | --- | --- |
| ***response to hypoxia*** | | |
| LmjF.28.0090 | oxygen-sensing adenylate cyclase | Supress oxadative stress |
| ***response to stimulus*** | | |
| LmjF.12.0520 | vacuolar ATP synthase subunit, putative | Membrane fusion and anti-inflammatory functions |
| LmjF.25.1460 | cAMP response protein, putative | Nuclear transcription factor |
| LmjF.28.0090 | oxygen-sensing adenylate cyclase | Supress oxadative stress |
| LmjF.28.2140 | A/G-specific adenine glycosylase, putative | DNA repair system after oxidative stress/damange |
| LmjF.30.0660 | pdz domain containing protein, putative | Candidate for increased pathogenicity |
| LmjF.35.0500 | proteophosphoglycan ppg3, putative | Surface protein - lipophosphoglycan (LPG) |
| LmjF.35.0550 | proteophosphoglycan ppg1 | Surface protein - lipophosphoglycan (LPG) |
| LmjF.35.1280 | 2OG-Fe(II) oxygenase superfamily, putative | Iron and Heme metabolism - cell grow |
| LmjF.35.1740 | ubiquitin hydrolase, putative | Parasite viability |
| LmjF.35.3450 | DNA-repair protein, putative | DNA repair system |
| LmjF.35.3460 | peroxidase, putative | Trypanothione pathway, converts hydrogen peroxide into water |
| LmjF.36.1950 | DNA mismatch repair protein MSH6, putative | DNA repair system after oxidative stress/damange |
| LmjF.36.1980 | serine/threonine protein phosphatase 2B catalytic subunit A2, putative | Intracellular signaling |
| LmjF.36.6347 | hypothetical protein, conserved |  |
| ***positive regulation of cellular component organization,*** | | |
| LmjF.25.0720, LmjF.25.0730 | eukaryotic initiation factor 5a, putative | Cell wall stabilitiy when stress/oxadative stress, functions as a regulator of apoptosis |
| ***translational elongation*** | | |
| LmjF.17.0080, LmjF.17.0081, LmjF.17.0082, LmjF.17.0083, LmjF.17.0084, LmjF.17.0085, LmjF.17.0086 | elongation factor 1-alpha | Function as an important virulence factor. |
| ***mitochondrial transmembrane transport*** | | |
| LmjF.01.0335 | Mitochondrial pyruvate carrier 1 | Oxidation phosphorylation, survival in the host |
| LmjF.28.2170 | Mitochondrial import receptor subunit ATOM69, putative |  |
| LmjF.35.3900 | hypothetical protein, conserved |  |
