# Supplementary material for: Genetic coping mechanisms observed in Leishmania tropica, from the Middle East region, enhance the survival of the parasite after drug exposure
Source: PLoS One. 2024 Dec 3;19(12):e0310821. doi: 10.1371/journal.pone.0310821 (PMC11614225; doi:10.1371/journal.pone.0310821)
Supplement: S4 Table — Bgd count: Number of genes with this term in the genome; Result count: Number of genes with this term in this analysis; Pct of bgd: Of the genes in the background with this term, the percent that is present in the result. Fold enrichment: The percent of genes with this term in this analysis divided by the percent of genes with this term in the genome. (DOCX) [file pone.0310821.s011.docx]

**S4 Table**

| ID | Name | Bgd count | Result count | Result gene list | Pct of bgd | Fold enrichment | Odds ratio | Benjamini |
| --- | --- | --- | --- | --- | --- | --- | --- | --- |
| GO:0006002 | fructose 6-phosphate metabolic process | 1 | 1 | LmjF.29.2510, | 100.0 | 148.05 | inf | 0.442893941814 |
| GO:0043486 | histone exchange | 2 | 1 | LmjF.11.0980, | 50.0 | 74.03 | 151.0 | 0.442893941814 |
| GO:0042743 | hydrogen peroxide metabolic process | 3 | 1 | LmjF.29.1160, | 33.3 | 49.35 | 75.49 | 0.442893941814 |
| GO:0042744 | hydrogen peroxide catabolic process | 3 | 1 | LmjF.29.1160, | 33.3 | 49.35 | 75.49 | 0.442893941814 |
| GO:0006743 | ubiquinone metabolic process | 4 | 1 | LmjF.29.2350, | 25.0 | 37.01 | 50.32 | 0.442893941814 |
| GO:0006744 | ubiquinone biosynthetic process | 4 | 1 | LmjF.29.2350, | 25.0 | 37.01 | 50.32 | 0.442893941814 |
| GO:0042026 | protein refolding | 4 | 1 | LmjF.32.1850, | 25.0 | 37.01 | 50.32 | 0.442893941814 |
| GO:0042181 | ketone biosynthetic process | 4 | 1 | LmjF.29.2350, | 25.0 | 37.01 | 50.32 | 0.442893941814 |
| GO:1901663 | quinone biosynthetic process | 4 | 1 | LmjF.29.2350, | 25.0 | 37.01 | 50.32 | 0.442893941814 |
| GO:0006383 | transcription by RNA polymerase III | 5 | 1 | LmjF.29.1320, | 20.0 | 29.61 | 37.73 | 0.451474369189 |
| GO:1901661 | quinone metabolic process | 5 | 1 | LmjF.29.2350, | 20.0 | 29.61 | 37.73 | 0.451474369189 |
| GO:0034728 | nucleosome organization | 6 | 1 | LmjF.11.0980, | 16.7 | 24.68 | 30.18 | 0.456918361574 |
| GO:0044458 | motile cilium assembly | 6 | 1 | LmjF.32.0730, | 16.7 | 24.68 | 30.18 | 0.456918361574 |
| GO:0042180 | cellular ketone metabolic process | 7 | 1 | LmjF.29.2350, | 14.3 | 21.15 | 25.14 | 0.493374908824 |
