# Supplementary material for: Genetic coping mechanisms observed in Leishmania tropica, from the Middle East region, enhance the survival of the parasite after drug exposure
Source: PLoS One. 2024 Dec 3;19(12):e0310821. doi: 10.1371/journal.pone.0310821 (PMC11614225; doi:10.1371/journal.pone.0310821)
Supplement: S5 Table — Bgd count: Number of genes with this term in the genome; Result count: Number of genes with this term in this analysis; Pct of bgd: Of the genes in the background with this term, the percent that is present in the result. Fold enrichment: The percent of genes with this term in this analysis divided by the percent of genes with this term in the genome. (DOCX) [file pone.0310821.s012.docx]

**S5 Table**

| Name | Bgd count | Result count | Result gene list | Pct of bgd | Fold enrichment | Odds ratio | Benjamini |
| --- | --- | --- | --- | --- | --- | --- | --- |
| DNA-templated transcription, elongation | 5 | 3 | LmjF.29.2550,LmjF.36.3980,LmjF.36.4145, | 60.0 | 7.64 | 17.71 | 0.517389832919 |
| regulation of cilium movement | 2 | 2 | LmjF.36.3490,LmjF.36.4500, | 100.0 | 12.73 | inf | 0.517389832919 |
| regulation of microtubule-based process | 2 | 2 | LmjF.36.3490,LmjF.36.4500, | 100.0 | 12.73 | inf | 0.517389832919 |
| acetyl-CoA biosynthetic process from acetate | 2 | 2 | LmjF.23.0540,LmjF.23.0710, | 100.0 | 12.73 | inf | 0.517389832919 |
| regulation of cellular component movement | 2 | 2 | LmjF.36.3490,LmjF.36.4500, | 100.0 | 12.73 | inf | 0.517389832919 |
| tryptophanyl-tRNA aminoacylation | 2 | 2 | LmjF.23.0300,LmjF.29.0060, | 100.0 | 12.73 | inf | 0.517389832919 |
| regulation of microtubule-based movement | 2 | 2 | LmjF.36.3490,LmjF.36.4500, | 100.0 | 12.73 | inf | 0.517389832919 |
| acetate metabolic process | 2 | 2 | LmjF.23.0540,LmjF.23.0710, | 100.0 | 12.73 | inf | 0.517389832919 |
| double-strand break repair via nonhomologous end joining | 3 | 2 | LmjF.29.1050,LmjF.36.2940, | 66.7 | 8.49 | 23.56 | 0.561647669261 |
| GDP-mannose metabolic process | 3 | 2 | LmjF.23.0110,LmjF.36.1960, | 66.7 | 8.49 | 23.56 | 0.561647669261 |
| acetyl-CoA biosynthetic process | 3 | 2 | LmjF.23.0540,LmjF.23.0710, | 66.7 | 8.49 | 23.56 | 0.561647669261 |
| calcineurin-mediated signaling | 3 | 2 | LmjF.12.0050,LmjF.36.1980, | 66.7 | 8.49 | 23.56 | 0.561647669261 |
| GDP-mannose biosynthetic process | 3 | 2 | LmjF.23.0110,LmjF.36.1960, | 66.7 | 8.49 | 23.56 | 0.561647669261 |
| negative regulation of nucleic acid-templated transcription | 3 | 2 | LmjF.23.1350,LmjF.36.1060, | 66.7 | 8.49 | 23.56 | 0.561647669261 |
| thioester biosynthetic process | 3 | 2 | LmjF.23.0540,LmjF.23.0710, | 66.7 | 8.49 | 23.56 | 0.561647669261 |
| negative regulation of RNA biosynthetic process | 3 | 2 | LmjF.23.1350,LmjF.36.1060, | 66.7 | 8.49 | 23.56 | 0.561647669261 |
| acyl-CoA biosynthetic process | 3 | 2 | LmjF.23.0540,LmjF.23.0710, | 66.7 | 8.49 | 23.56 | 0.561647669261 |
| transcription elongation from RNA polymerase II promoter | 3 | 2 | LmjF.29.2550,LmjF.36.3980, | 66.7 | 8.49 | 23.56 | 0.561647669261 |
| negative regulation of transcription, DNA-templated | 3 | 2 | LmjF.23.1350,LmjF.36.1060, | 66.7 | 8.49 | 23.56 | 0.561647669261 |
| regulation of localization | 8 | 3 | LmjF.29.1230,LmjF.36.3490,LmjF.36.4500, | 37.5 | 4.77 | 7.08 | 0.561647669261 |
| cellular response to stimulus | 147 | 19 | LmjF.12.0050,LmjF.23.1260,LmjF.23.1330,LmjF.29.1050,LmjF.29.1450,LmjF.29.1580,LmjF.29.1710,LmjF.29.2160,LmjF.29.2260,LmjF.29.2440,LmjF.29.2590,LmjF.36.0100,LmjF.36.0750,LmjF.36.1820,LmjF.36.1900,LmjF.36.1950,LmjF.36.1980,LmjF.36.2620,LmjF.36.2940, | 12.9 | 1.65 | 1.77 | 0.561647669261 |
| signaling | 65 | 10 | LmjF.12.0050,LmjF.23.1260,LmjF.29.1450,LmjF.29.2020,LmjF.29.2160,LmjF.29.2440,LmjF.29.2590,LmjF.36.1820,LmjF.36.1900,LmjF.36.1980, | 15.4 | 1.96 | 2.16 | 0.561647669261 |
| regulation of macromolecule biosynthetic process | 56 | 9 | LmjF.12.0350,LmjF.23.1350,LmjF.29.1780,LmjF.29.2550,LmjF.36.1060,LmjF.36.3880,LmjF.36.3980,LmjF.36.4145,LmjF.36.4490, | 16.1 | 2.05 | 2.27 | 0.561647669261 |
| regulation of cellular macromolecule biosynthetic process | 56 | 9 | LmjF.12.0350,LmjF.23.1350,LmjF.29.1780,LmjF.29.2550,LmjF.36.1060,LmjF.36.3880,LmjF.36.3980,LmjF.36.4145,LmjF.36.4490, | 16.1 | 2.05 | 2.27 | 0.561647669261 |
| nucleic acid metabolic process | 445 | 46 | LmjF.12.0250,LmjF.12.0560,LmjF.12.1305,LmjF.23.0030,LmjF.23.0050,LmjF.23.0300,LmjF.23.0543,LmjF.23.1330,LmjF.23.1350,LmjF.23.1550,LmjF.23.1770,LmjF.29.0060,LmjF.29.0140,LmjF.29.0680,LmjF.29.0900,LmjF.29.1050,LmjF.29.1320,LmjF.29.1580,LmjF.29.1710,LmjF.29.2260,LmjF.29.2550,LmjF.36.0100,LmjF.36.0470,LmjF.36.0535,LmjF.36.0640,LmjF.36.0700,LmjF.36.0750,LmjF.36.0920,LmjF.36.1050,LmjF.36.1300,LmjF.36.1790,LmjF.36.1950,LmjF.36.2170,LmjF.36.2370,LmjF.36.2500,LmjF.36.2620,LmjF.36.2940,LmjF.36.3200,LmjF.36.3840,LmjF.36.3930,LmjF.36.3980,LmjF.36.4145,LmjF.36.4190,LmjF.36.4210,LmjF.36.4280,LmjF.36.5620, | 10.3 | 1.32 | 1.39 | 0.561647669261 |
| regulation of cellular biosynthetic process | 57 | 9 | LmjF.12.0350,LmjF.23.1350,LmjF.29.1780,LmjF.29.2550,LmjF.36.1060,LmjF.36.3880,LmjF.36.3980,LmjF.36.4145,LmjF.36.4490, | 15.8 | 2.01 | 2.22 | 0.561647669261 |
| regulation of biosynthetic process | 57 | 9 | LmjF.12.0350,LmjF.23.1350,LmjF.29.1780,LmjF.29.2550,LmjF.36.1060,LmjF.36.3880,LmjF.36.3980,LmjF.36.4145,LmjF.36.4490, | 15.8 | 2.01 | 2.22 | 0.561647669261 |
| signal transduction | 57 | 9 | LmjF.12.0050,LmjF.23.1260,LmjF.29.1450,LmjF.29.2160,LmjF.29.2440,LmjF.29.2590,LmjF.36.1820,LmjF.36.1900,LmjF.36.1980, | 15.8 | 2.01 | 2.22 | 0.561647669261 |
| double-strand break repair | 10 | 3 | LmjF.29.1050,LmjF.29.2260,LmjF.36.2940, | 30.0 | 3.82 | 5.05 | 0.561647669261 |
| cell communication | 68 | 10 | LmjF.12.0050,LmjF.23.1260,LmjF.29.1450,LmjF.29.2020,LmjF.29.2160,LmjF.29.2440,LmjF.29.2590,LmjF.36.1820,LmjF.36.1900,LmjF.36.1980, | 14.7 | 1.87 | 2.05 | 0.561647669261 |
| DNA metabolic process | 137 | 17 | LmjF.12.0640,LmjF.23.1330,LmjF.23.1350,LmjF.29.1050,LmjF.29.1580,LmjF.29.1710,LmjF.29.2260,LmjF.36.0100,LmjF.36.0750,LmjF.36.1950,LmjF.36.2370,LmjF.36.2620,LmjF.36.2940,LmjF.36.3200,LmjF.36.3930,LmjF.36.4190,LmjF.36.6710, | 12.4 | 1.58 | 1.69 | 0.561647669261 |
| regulation of transcription, DNA-templated | 33 | 6 | LmjF.12.0350,LmjF.23.1350,LmjF.29.2550,LmjF.36.1060,LmjF.36.3980,LmjF.36.4145, | 18.2 | 2.31 | 2.63 | 0.561647669261 |
| regulation of nucleic acid-templated transcription | 33 | 6 | LmjF.12.0350,LmjF.23.1350,LmjF.29.2550,LmjF.36.1060,LmjF.36.3980,LmjF.36.4145, | 18.2 | 2.31 | 2.63 | 0.561647669261 |
| regulation of RNA biosynthetic process | 33 | 6 | LmjF.12.0350,LmjF.23.1350,LmjF.29.2550,LmjF.36.1060,LmjF.36.3980,LmjF.36.4145, | 18.2 | 2.31 | 2.63 | 0.561647669261 |
| amino acid activation | 25 | 5 | LmjF.12.0250,LmjF.23.0300,LmjF.29.0060,LmjF.36.3840,LmjF.36.5620, | 20.0 | 2.55 | 2.95 | 0.561647669261 |
| tRNA aminoacylation for protein translation | 25 | 5 | LmjF.12.0250,LmjF.23.0300,LmjF.29.0060,LmjF.36.3840,LmjF.36.5620, | 20.0 | 2.55 | 2.95 | 0.561647669261 |
| tRNA aminoacylation | 25 | 5 | LmjF.12.0250,LmjF.23.0300,LmjF.29.0060,LmjF.36.3840,LmjF.36.5620, | 20.0 | 2.55 | 2.95 | 0.561647669261 |
| nucleic acid-templated transcription | 42 | 7 | LmjF.12.0560,LmjF.29.0140,LmjF.29.1320,LmjF.29.2550,LmjF.36.2500,LmjF.36.3980,LmjF.36.4145, | 16.7 | 2.12 | 2.37 | 0.561647669261 |
| transcription, DNA-templated | 42 | 7 | LmjF.12.0560,LmjF.29.0140,LmjF.29.1320,LmjF.29.2550,LmjF.36.2500,LmjF.36.3980,LmjF.36.4145, | 16.7 | 2.12 | 2.37 | 0.561647669261 |
| response to stimulus | 267 | 29 | LmjF.12.0050,LmjF.12.0520,LmjF.23.0210,LmjF.23.0220,LmjF.23.0250,LmjF.23.0340,LmjF.23.1260,LmjF.23.1330,LmjF.23.1510,LmjF.29.1050,LmjF.29.1450,LmjF.29.1580,LmjF.29.1600,LmjF.29.1710,LmjF.29.2020,LmjF.29.2160,LmjF.29.2260,LmjF.29.2440,LmjF.29.2590,LmjF.36.0100,LmjF.36.0750,LmjF.36.1820,LmjF.36.1900,LmjF.36.1950,LmjF.36.1980,LmjF.36.2620,LmjF.36.2940,LmjF.36.3010,LmjF.36.4530, | 10.9 | 1.38 | 1.46 | 0.561647669261 |
| regulation of primary metabolic process | 99 | 13 | LmjF.12.0350,LmjF.23.0760,LmjF.23.1350,LmjF.23.1460,LmjF.29.0120,LmjF.29.1780,LmjF.29.2550,LmjF.36.0860,LmjF.36.1060,LmjF.36.3880,LmjF.36.3980,LmjF.36.4145,LmjF.36.4490, | 13.1 | 1.67 | 1.8 | 0.561647669261 |
| regulation of nitrogen compound metabolic process | 100 | 13 | LmjF.12.0350,LmjF.23.0760,LmjF.23.1350,LmjF.23.1460,LmjF.29.0120,LmjF.29.1780,LmjF.29.2550,LmjF.36.0860,LmjF.36.1060,LmjF.36.3880,LmjF.36.3980,LmjF.36.4145,LmjF.36.4490, | 13.0 | 1.65 | 1.78 | 0.561647669261 |
