# Supplementary material for: Genetic coping mechanisms observed in Leishmania tropica, from the Middle East region, enhance the survival of the parasite after drug exposure
Source: PLoS One. 2024 Dec 3;19(12):e0310821. doi: 10.1371/journal.pone.0310821 (PMC11614225; doi:10.1371/journal.pone.0310821)
Supplement: S6 Table — Bgd count: Number of genes with this term in the genome; Result count: Number of genes with this term in this analysis; Pct of bgd: Of the genes in the background with this term, the percent that is present in the result. Fold enrichment: The percent of genes with this term in this analysis divided by the percent of genes with this term in the genome. (DOCX) [file pone.0310821.s013.docx]

**S6 Table**

| ID | Name | Bgd count | Result count | Result gene list | Pct of bgd | Fold enrichment | Odds ratio | Benjamini |
| --- | --- | --- | --- | --- | --- | --- | --- | --- |
| GO:0006414 | translational elongation | 22 | 7 | LmjF.17.0080,LmjF.17.0081,LmjF.17.0082,LmjF.17.0083,LmjF.17.0084,LmjF.17.0085,LmjF.17.0086, | 31.8 | 10.88 | 16.14 | 0.000830286659266 |
| GO:0045905 | positive regulation of translational termination | 2 | 2 | LmjF.25.0720,LmjF.25.0730, | 100.0 | 34.2 | inf | 0.0620451044906 |
| GO:0043243 | positive regulation of protein-containing complex disassembly | 2 | 2 | LmjF.25.0720,LmjF.25.0730, | 100.0 | 34.2 | inf | 0.0620451044906 |
| GO:0045901 | positive regulation of translational elongation | 2 | 2 | LmjF.25.0720,LmjF.25.0730, | 100.0 | 34.2 | inf | 0.0620451044906 |
| GO:0006449 | regulation of translational termination | 2 | 2 | LmjF.25.0720,LmjF.25.0730, | 100.0 | 34.2 | inf | 0.0620451044906 |
| GO:0051130 | positive regulation of cellular component organization | 2 | 2 | LmjF.25.0720,LmjF.25.0730, | 100.0 | 34.2 | inf | 0.0620451044906 |
| GO:0043244 | regulation of protein-containing complex disassembly | 3 | 2 | LmjF.25.0720,LmjF.25.0730, | 66.7 | 22.8 | 67.2 | 0.121699769453 |
| GO:0045727 | positive regulation of translation | 3 | 2 | LmjF.25.0720,LmjF.25.0730, | 66.7 | 22.8 | 67.2 | 0.121699769453 |
| GO:0034250 | positive regulation of cellular amide metabolic process | 3 | 2 | LmjF.25.0720,LmjF.25.0730, | 66.7 | 22.8 | 67.2 | 0.121699769453 |
| GO:0006955 | immune response | 4 | 2 | LmjF.35.0500,LmjF.35.0550, | 50.0 | 17.1 | 33.6 | 0.134280929356 |
| GO:0050776 | regulation of immune response | 4 | 2 | LmjF.35.0500,LmjF.35.0550, | 50.0 | 17.1 | 33.6 | 0.134280929356 |
| GO:0032270 | positive regulation of cellular protein metabolic process | 4 | 2 | LmjF.25.0720,LmjF.25.0730, | 50.0 | 17.1 | 33.6 | 0.134280929356 |
| GO:0002764 | immune response-regulating signaling pathway | 4 | 2 | LmjF.35.0500,LmjF.35.0550, | 50.0 | 17.1 | 33.6 | 0.134280929356 |
| GO:0002224 | toll-like receptor signaling pathway | 4 | 2 | LmjF.35.0500,LmjF.35.0550, | 50.0 | 17.1 | 33.6 | 0.134280929356 |
| GO:0002221 | pattern recognition receptor signaling pathway | 4 | 2 | LmjF.35.0500,LmjF.35.0550, | 50.0 | 17.1 | 33.6 | 0.134280929356 |
| GO:0051247 | positive regulation of protein metabolic process | 4 | 2 | LmjF.25.0720,LmjF.25.0730, | 50.0 | 17.1 | 33.6 | 0.134280929356 |
| GO:0006448 | regulation of translational elongation | 5 | 2 | LmjF.25.0720,LmjF.25.0730, | 40.0 | 13.68 | 22.39 | 0.195120337814 |
| GO:0002682 | regulation of immune system process | 5 | 2 | LmjF.35.0500,LmjF.35.0550, | 40.0 | 13.68 | 22.39 | 0.195120337814 |
| GO:0030148 | sphingolipid biosynthetic process | 6 | 2 | LmjF.26.1670,LmjF.26.1680, | 33.3 | 11.4 | 16.79 | 0.241641840451 |
| GO:0002376 | immune system process | 6 | 2 | LmjF.35.0500,LmjF.35.0550, | 33.3 | 11.4 | 16.79 | 0.241641840451 |
| GO:0006665 | sphingolipid metabolic process | 6 | 2 | LmjF.26.1670,LmjF.26.1680, | 33.3 | 11.4 | 16.79 | 0.241641840451 |
| GO:0009987 | cellular process | 2323 | 82 | LmjF.01.0310,LmjF.01.0320,LmjF.01.0335,LmjF.06.0570,LmjF.06.1270,LmjF.12.0520,LmjF.12.0530,LmjF.15.1010,LmjF.17.0080,LmjF.17.0081,LmjF.17.0082,LmjF.17.0083,LmjF.17.0084,LmjF.17.0085,LmjF.17.0086,LmjF.19.0220,LmjF.22.0790,LmjF.23.1165,LmjF.23.1220,LmjF.24.0670,LmjF.24.1315,LmjF.24.1320,LmjF.25.1450,LmjF.25.2180,LmjF.26.1000,LmjF.26.1020,LmjF.26.1670,LmjF.26.1680,LmjF.26.1730,LmjF.28.0040,LmjF.28.0050,LmjF.28.0090,LmjF.28.0110,LmjF.28.0140,LmjF.28.0150,LmjF.28.0170,LmjF.28.0780,LmjF.28.0790,LmjF.28.2060,LmjF.28.2080,LmjF.28.2110,LmjF.28.2120,LmjF.28.2130,LmjF.28.2140,LmjF.28.2170,LmjF.30.0660,LmjF.30.0670,LmjF.30.0680,LmjF.30.0690,LmjF.30.0730,LmjF.30.0735,LmjF.30.0780,LmjF.30.0800,LmjF.33.1340,LmjF.33.1350,LmjF.33.1355,LmjF.34.2540,LmjF.35.0500,LmjF.35.0550,LmjF.35.0860,LmjF.35.1200,LmjF.35.1250,LmjF.35.1280,LmjF.35.1740,LmjF.35.1750,LmjF.35.1790,LmjF.35.3450,LmjF.35.3900,LmjF.35.4700,LmjF.36.0510,LmjF.36.0530,LmjF.36.0535,LmjF.36.0550,LmjF.36.1950,LmjF.36.1960,LmjF.36.1980,LmjF.36.2720,LmjF.36.3030,LmjF.36.3050,LmjF.36.3060,LmjF.36.3070,LmjF.36.3680, | 3.5 | 1.21 | 1.43 | 0.241641840451 |
| GO:1990542 | mitochondrial transmembrane transport | 20 | 3 | LmjF.01.0335,LmjF.28.2170,LmjF.35.3900, | 15.0 | 5.13 | 5.95 | 0.241641840451 |
| GO:0019941 | modification-dependent protein catabolic process | 54 | 5 | LmjF.28.0110,LmjF.28.2110,LmjF.28.2120,LmjF.35.1740,LmjF.36.3060, | 9.3 | 3.17 | 3.46 | 0.241641840451 |
| GO:0006511 | ubiquitin-dependent protein catabolic process | 54 | 5 | LmjF.28.0110,LmjF.28.2110,LmjF.28.2120,LmjF.35.1740,LmjF.36.3060, | 9.3 | 3.17 | 3.46 | 0.241641840451 |
| GO:0043632 | modification-dependent macromolecule catabolic process | 54 | 5 | LmjF.28.0110,LmjF.28.2110,LmjF.28.2120,LmjF.35.1740,LmjF.36.3060, | 9.3 | 3.17 | 3.46 | 0.241641840451 |
| GO:0050896 | response to stimulus | 267 | 14 | LmjF.12.0520,LmjF.25.1460,LmjF.28.0090,LmjF.28.2140,LmjF.30.0660,LmjF.35.0500,LmjF.35.0550,LmjF.35.1280,LmjF.35.1740,LmjF.35.3450,LmjF.35.3460,LmjF.36.1950,LmjF.36.1980,LmjF.36.6347, | 5.2 | 1.79 | 1.91 | 0.241641840451 |
| GO:0044271 | cellular nitrogen compound biosynthetic process | 402 | 19 | LmjF.06.0570,LmjF.06.1270,LmjF.17.0080,LmjF.17.0081,LmjF.17.0082,LmjF.17.0083,LmjF.17.0084,LmjF.17.0085,LmjF.17.0086,LmjF.22.0790,LmjF.25.1450,LmjF.28.0090,LmjF.28.0140,LmjF.28.2060,LmjF.30.0670,LmjF.30.0680,LmjF.30.0690,LmjF.30.0780,LmjF.36.1960, | 4.7 | 1.62 | 1.73 | 0.241641840451 |
| GO:0007165 | signal transduction | 57 | 5 | LmjF.28.0090,LmjF.30.0660,LmjF.35.0500,LmjF.35.0550,LmjF.36.1980, | 8.8 | 3.0 | 3.26 | 0.241641840451 |
| GO:0019319 | hexose biosynthetic process | 9 | 2 | LmjF.12.0530,LmjF.36.1960, | 22.2 | 7.6 | 9.59 | 0.241641840451 |
| GO:0051716 | cellular response to stimulus | 147 | 9 | LmjF.28.0090,LmjF.28.2140,LmjF.30.0660,LmjF.35.0500,LmjF.35.0550,LmjF.35.1280,LmjF.35.3450,LmjF.36.1950,LmjF.36.1980, | 6.1 | 2.09 | 2.23 | 0.241641840451 |
| GO:0010628 | positive regulation of gene expression | 23 | 3 | LmjF.25.0720,LmjF.25.0730,LmjF.28.0040, | 13.0 | 4.46 | 5.05 | 0.241641840451 |
| GO:0017038 | protein import | 23 | 3 | LmjF.28.2170,LmjF.35.3900,LmjF.36.2720, | 13.0 | 4.46 | 5.05 | 0.241641840451 |
| GO:0043649 | dicarboxylic acid catabolic process | 1 | 1 | LmjF.15.1010, | 100.0 | 34.2 | inf | 0.241641840451 |
| GO:0045761 | regulation of adenylate cyclase activity | 1 | 1 | LmjF.28.0090, | 100.0 | 34.2 | inf | 0.241641840451 |
| GO:0052652 | cyclic purine nucleotide metabolic process | 1 | 1 | LmjF.28.0090, | 100.0 | 34.2 | inf | 0.241641840451 |
| GO:0070482 | response to oxygen levels | 1 | 1 | LmjF.28.0090, | 100.0 | 34.2 | inf | 0.241641840451 |
| GO:0046058 | cAMP metabolic process | 1 | 1 | LmjF.28.0090, | 100.0 | 34.2 | inf | 0.241641840451 |
| GO:0036293 | response to decreased oxygen levels | 1 | 1 | LmjF.28.0090, | 100.0 | 34.2 | inf | 0.241641840451 |
| GO:0051339 | regulation of lyase activity | 1 | 1 | LmjF.28.0090, | 100.0 | 34.2 | inf | 0.241641840451 |
| GO:0000380 | alternative mRNA splicing, via spliceosome | 1 | 1 | LmjF.36.0535, | 100.0 | 34.2 | inf | 0.241641840451 |
| GO:0031279 | regulation of cyclase activity | 1 | 1 | LmjF.28.0090, | 100.0 | 34.2 | inf | 0.241641840451 |
| GO:0001666 | response to hypoxia | 1 | 1 | LmjF.28.0090, | 100.0 | 34.2 | inf | 0.241641840451 |
| GO:0001731 | formation of translation preinitiation complex | 1 | 1 | LmjF.22.0790, | 100.0 | 34.2 | inf | 0.241641840451 |
| GO:0002183 | cytoplasmic translational initiation | 1 | 1 | LmjF.22.0790, | 100.0 | 34.2 | inf | 0.241641840451 |
| GO:0003032 | detection of oxygen | 1 | 1 | LmjF.28.0090, | 100.0 | 34.2 | inf | 0.241641840451 |
| GO:0006103 | 2-oxoglutarate metabolic process | 1 | 1 | LmjF.15.1010, | 100.0 | 34.2 | inf | 0.241641840451 |
| GO:0006171 | cAMP biosynthetic process | 1 | 1 | LmjF.28.0090, | 100.0 | 34.2 | inf | 0.241641840451 |
| GO:0006491 | N-glycan processing | 1 | 1 | LmjF.26.1000, | 100.0 | 34.2 | inf | 0.241641840451 |
| GO:0006538 | glutamate catabolic process | 1 | 1 | LmjF.15.1010, | 100.0 | 34.2 | inf | 0.241641840451 |
| GO:0019551 | glutamate catabolic process to 2-oxoglutarate | 1 | 1 | LmjF.15.1010, | 100.0 | 34.2 | inf | 0.241641840451 |
| GO:0070483 | detection of hypoxia | 1 | 1 | LmjF.28.0090, | 100.0 | 34.2 | inf | 0.241641840451 |
| GO:0009593 | detection of chemical stimulus | 1 | 1 | LmjF.28.0090, | 100.0 | 34.2 | inf | 0.241641840451 |
| GO:0050794 | regulation of cellular process | 224 | 12 | LmjF.24.0670,LmjF.25.0720,LmjF.25.0730,LmjF.26.1730,LmjF.28.0040,LmjF.28.0090,LmjF.30.0660,LmjF.35.0500,LmjF.35.0550,LmjF.35.1250,LmjF.36.0550,LmjF.36.1980, | 5.4 | 1.83 | 1.95 | 0.246616167669 |
| GO:0010557 | positive regulation of macromolecule biosynthetic process | 10 | 2 | LmjF.25.0720,LmjF.25.0730, | 20.0 | 6.84 | 8.39 | 0.246965810115 |
| GO:0031328 | positive regulation of cellular biosynthetic process | 10 | 2 | LmjF.25.0720,LmjF.25.0730, | 20.0 | 6.84 | 8.39 | 0.246965810115 |
| GO:0009891 | positive regulation of biosynthetic process | 10 | 2 | LmjF.25.0720,LmjF.25.0730, | 20.0 | 6.84 | 8.39 | 0.246965810115 |
| GO:0046364 | monosaccharide biosynthetic process | 10 | 2 | LmjF.12.0530,LmjF.36.1960, | 20.0 | 6.84 | 8.39 | 0.246965810115 |
| GO:0006412 | translation | 253 | 13 | LmjF.06.0570,LmjF.17.0080,LmjF.17.0081,LmjF.17.0082,LmjF.17.0083,LmjF.17.0084,LmjF.17.0085,LmjF.17.0086,LmjF.22.0790,LmjF.25.1450,LmjF.30.0670,LmjF.30.0680,LmjF.30.0690, | 5.1 | 1.76 | 1.87 | 0.246965810115 |
| GO:0008152 | metabolic process | 1867 | 66 | LmjF.01.0310,LmjF.01.0320,LmjF.06.0570,LmjF.06.1270,LmjF.12.0530,LmjF.15.1010,LmjF.17.0080,LmjF.17.0081,LmjF.17.0082,LmjF.17.0083,LmjF.17.0084,LmjF.17.0085,LmjF.17.0086,LmjF.19.0220,LmjF.22.0770,LmjF.22.0780,LmjF.22.0790,LmjF.24.0670,LmjF.24.1315,LmjF.25.1450,LmjF.26.1000,LmjF.26.1670,LmjF.26.1680,LmjF.26.1730,LmjF.28.0040,LmjF.28.0090,LmjF.28.0110,LmjF.28.0140,LmjF.28.0170,LmjF.28.0780,LmjF.28.0790,LmjF.28.2060,LmjF.28.2080,LmjF.28.2100,LmjF.28.2110,LmjF.28.2120,LmjF.28.2140,LmjF.30.0660,LmjF.30.0670,LmjF.30.0680,LmjF.30.0690,LmjF.30.0780,LmjF.30.0800,LmjF.30.1610,LmjF.33.1330,LmjF.33.1355,LmjF.35.0860,LmjF.35.1200,LmjF.35.1220,LmjF.35.1230,LmjF.35.1250,LmjF.35.1280,LmjF.35.1740,LmjF.35.1790,LmjF.35.3450,LmjF.35.3460,LmjF.36.0530,LmjF.36.0535,LmjF.36.0550,LmjF.36.1950,LmjF.36.1960,LmjF.36.1970,LmjF.36.2710,LmjF.36.3060,LmjF.36.3070,LmjF.36.3680, | 3.5 | 1.21 | 1.36 | 0.252301264869 |
| GO:0043043 | peptide biosynthetic process | 255 | 13 | LmjF.06.0570,LmjF.17.0080,LmjF.17.0081,LmjF.17.0082,LmjF.17.0083,LmjF.17.0084,LmjF.17.0085,LmjF.17.0086,LmjF.22.0790,LmjF.25.1450,LmjF.30.0670,LmjF.30.0680,LmjF.30.0690, | 5.1 | 1.74 | 1.85 | 0.252301264869 |
| GO:0016051 | carbohydrate biosynthetic process | 11 | 2 | LmjF.12.0530,LmjF.36.1960, | 18.2 | 6.22 | 7.46 | 0.277665018899 |
| GO:0043604 | amide biosynthetic process | 260 | 13 | LmjF.06.0570,LmjF.17.0080,LmjF.17.0081,LmjF.17.0082,LmjF.17.0083,LmjF.17.0084,LmjF.17.0085,LmjF.17.0086,LmjF.22.0790,LmjF.25.1450,LmjF.30.0670,LmjF.30.0680,LmjF.30.0690, | 5.0 | 1.71 | 1.81 | 0.278126378296 |
| GO:0023052 | signaling | 65 | 5 | LmjF.28.0090,LmjF.30.0660,LmjF.35.0500,LmjF.35.0550,LmjF.36.1980, | 7.7 | 2.63 | 2.82 | 0.278126378296 |
| GO:0044257 | cellular protein catabolic process | 67 | 5 | LmjF.28.0110,LmjF.28.2110,LmjF.28.2120,LmjF.35.1740,LmjF.36.3060, | 7.5 | 2.55 | 2.73 | 0.301185439469 |
| GO:0051603 | proteolysis involved in cellular protein catabolic process | 67 | 5 | LmjF.28.0110,LmjF.28.2110,LmjF.28.2120,LmjF.35.1740,LmjF.36.3060, | 7.5 | 2.55 | 2.73 | 0.301185439469 |
| GO:0006518 | peptide metabolic process | 266 | 13 | LmjF.06.0570,LmjF.17.0080,LmjF.17.0081,LmjF.17.0082,LmjF.17.0083,LmjF.17.0084,LmjF.17.0085,LmjF.17.0086,LmjF.22.0790,LmjF.25.1450,LmjF.30.0670,LmjF.30.0680,LmjF.30.0690, | 4.9 | 1.67 | 1.77 | 0.303429830276 |
| GO:0006470 | protein dephosphorylation | 28 | 3 | LmjF.28.0170,LmjF.28.0790,LmjF.36.0530, | 10.7 | 3.66 | 4.04 | 0.303429830276 |
| GO:0007154 | cell communication | 68 | 5 | LmjF.28.0090,LmjF.30.0660,LmjF.35.0500,LmjF.35.0550,LmjF.36.1980, | 7.4 | 2.51 | 2.69 | 0.303905913365 |
